# Achieving Molecular Sieving of CO_2_ from CH_4_ by Controlled Dynamical Movement and Host–Guest Interactions in Ultramicroporous VOFFIVE-1-Ni by Pillar Substitution

**DOI:** 10.1021/acs.nanolett.4c01305

**Published:** 2024-05-30

**Authors:** Ribooga Chang, Zoltán Bacsik, Guojun Zhou, Maria Strømme, Zhehao Huang, Michelle Åhlén, Ocean Cheung

**Affiliations:** † Division of Nanotechnology and Functional Materials, Department of Materials Science and Engineering, The Ångström Laboratory, Uppsala University, Box 35, SE-751 03, Uppsala, Sweden; ‡ Department of Materials and Environmental Chemistry, Stockholm University, SE-106 91, Stockholm, Sweden

**Keywords:** hybrid ultramicroporous materials, metal−organic frameworks, carbon capture, adsorption, separation

## Abstract

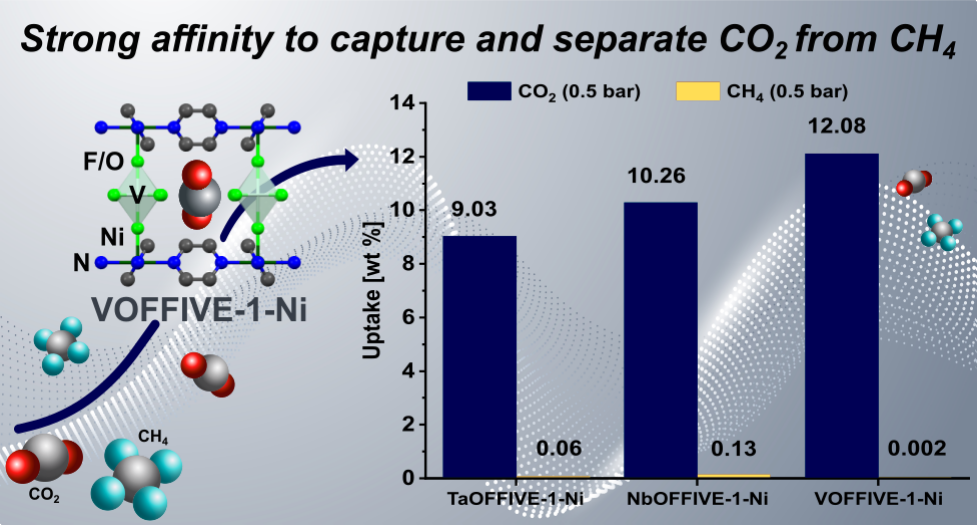

Engineering the building blocks in metal–organic materials is an effective strategy for tuning their dynamical properties and can affect their response to external guest molecules. Tailoring the interaction and diffusion of molecules into these structures is highly important, particularly for applications related to gas separation. Herein, we report a vanadium-based hybrid ultramicroporous material, VOFFIVE-1-Ni, with temperature-dependent dynamical properties and a strong affinity to effectively capture and separate carbon dioxide (CO_2_) from methane (CH_4_). VOFFIVE-1-Ni exhibits a CO_2_ uptake of 12.08 wt % (2.75 mmol g^–1^), a negligible CH_4_ uptake at 293 K (0.5 bar), and an excellent CO_2_-over-CH_4_ uptake ratio of 2280, far exceeding that of similar materials. The material also exhibits a favorable CO_2_ enthalpy of adsorption below −50 kJ mol^–1^, as well as fast CO_2_ adsorption rates (90% uptake reached within 20 s) that render the hydrolytically stable VOFFIVE-1-Ni a promising sorbent for applications such as biogas upgrading.

Gas separation and purification processes play a pivotal role in the manufacturing of many bulk chemicals for fuels, polymers, and plastics.^1,2^ In particular, the separation of CO_2_ from other gaseous species such as nitrogen (N_2_) or CH_4_ is critical not only for biogas upgrading^3,4^ but also in CO_2_ sequestration technologies. Several approaches for CO_2_ separation from N_2_ and/or CH_4_ have been reported and include techniques such as cryogenic distillation,^5^ chemical absorption,^5−8^ membrane separation,^5,6^ and physical adsorption.^5,6,8^ Solid adsorbents, in particular metal–organic materials (MOMs), have shown great promise as cost-effective and efficient alternatives due to their highly diverse structures which can be tuned through a careful selection of the materials’ inherent building units. Hybrid ultramicroporous materials (HUMs), a class of physisorbent, possess strong electrostatics as well as pore dimensions in the ultramicroporous range (<7 Å)^9^ which arise from the composition of the frameworks—the interconnection between 2D metal–organic square lattices (e.g., Ni(pyrazine)_2_) and anionic pillars (e.g., (SiF_6_)^2–^, (NbOF_5_)^2–^, (TaOF_5_)^2–^)^10−14^ which creates a 3D network structure. The unique pore geometry and surface functionality in HUMs result in the formation of strong adsorption sites, particularly for small polarizable molecules such as CO_2_ (3.3 Å).^15^ An excellent adsorption capacity of CO_2_ in the low-pressure range was observed in the prototypical HUM SIFSIX-3-Ni (SIFSIX = (SiF_6_)^2–^, 3 = pyrazine or pyz, and Ni = Ni^2+^) in which the material adsorbed approximately 0.39 mmol g^–1^ CO_2_ at 400 ppm (293 K).^11^ However, the inherent poor long-term stability of the material in the presence of water vapor limited the application of the HUM for gas capture and separation at ambient conditions.^16^ Substituting the anionic (SiF_6_)^2–^-pillar in the pyz-based HUM with the nucleophilic (NbOF_5_)^2–^ was therefore carried out in order to solve the long-term water stability issue of SIFSIX-3-Ni. The large atomic radius of Nb^5+^ significantly increased the bulkiness of the (NbOF_5_)^2–^-pillar compared to (SiF_6_)^2–^, and its increased nucleophilicity furthermore enhanced the material’s chemical and hydrolytic stability while preserving the framework structure.^17^ The resulting NbOFFIVE-1-Ni (NbOFFIVE = (NbOF_5_)^2–^ and 1 = pyz)^17,18^ material displayed excellent chemical and hydrolytic stability at 0–95% humidity as well as a contracted pore aperture due to the elongated Nb–F bond and tilted position of pyz-ligand,^17^ which enhanced the material’s CO_2_ sorption properties that is estimated to be approximately 1.3 mmol g^–1^ at 400 ppm. Furthermore, the increased rigidity of the HUM structure that arose from the limited rotational freedom of the ligand^18^ was successfully utilized to sieve propane (C_3_H_8_, <5.11 Å)^19^ from propylene (C_3_H_6_, 4.68 Å).^19^ Moreover, the substitution of the pyz-ligand in NbOFFIVE-1-Ni with the elongated 4,4′-bipyridine (bpy) molecule resulted in the formation of NbOFFIVE-bpy-Ni, which exhibited an excellent separation performance of xylene isomers due to the larger pore size and dynamic guest-responsive properties of the structure.^20^ Substitution of the building blocks, more specifically, the anionic pillar in HUMs can therefore be seen as a promising strategy for tailoring the pore structure of these materials on a subnanometer scale. In this work, we explore the substitution of (NbOF_5_)^2–^ in NbOFFIVE-1-Ni with two other anionic pillars, namely, (TaOF_5_)^2–^ and (VOF_5_)^2–^, and investigate their sorption properties for CO_2_ and their potential as molecular sieves for the separation of CO_2_ from CH_4_. Two isoreticular HUMs based on NbOFFIVE-1-Ni were hydrothermally synthesized using the anionic pillars (TaOF_5_)^2–^ ^14^ or (VOF_5_)^2–^. Substitution of the pyz-ligand with 2-aminopyrazine (pyz-NH_2_) was also carried out in NbOFFIVE-1-Ni in order to investigate the effects of linker substitution (please see page S-2 for details regarding synthesis). Three-dimensional electron diffraction (3D ED) (Figure S1 and Tables S1 and S2) was employed to study the structure of the pyz-NH_2_-based NbOFFIVE-2-Ni ([Ni(pyz-NH_2_)_2_(NbOF_5_)]_*n*_) (2 = pyz-NH_2_), due to the material’s small crystal size, and showed that the material crystallized in a tetragonal *P*4/*nbm* space group. Powder X-ray diffraction (PXRD) patterns of the as-synthesized pyz-based HUMs, namely VOFFIVE-1-Ni ([Ni(pyz)_2_(VOF_5_)·2H_2_O]_*n*_) and TaOFFIVE-1-Ni ([Ni(pyz)_2_(TaOF_5_)·CH_3_OH]_*n*_) were indexed (Figures S4–S6 and Table S3) to the same tetragonal crystal system and *I*4/*mcm* space group as NbOFFIVE-1-Ni ([Ni(pyz)_2_(NbOF_5_)·CH_3_OH]_*n*_) (Figure S5 and Table S3). The unit cell volume of the HUMs was reduced as the (TaOF_5_)^2–^ pillaring unit was substituted by (VOF_5_)^2–^ (Table S3) due to the increased electronegativity of the early-transition-metal cation (1.50, 1.60, and 1.63 for Ta, Nb, and V, respectively).^21^ In particular, the smaller unit cell constants of VOFFIVE-1-Ni (*a* = *b* = 9.883 Å, *c* = 15.092 Å), as compared to those of NbOFFIVE-1-Ni (*a* = *b* = 9.921 Å, *c* = 15.762 Å) and TaOFFIVE-1-Ni (*a* = *b* = 9.925 Å, *c* = 15.763 Å), was found to be most pronounced along the *c*-axis due to the influence of the (VOF_5_)^2–^ pillaring unit, leading to a shortening of the pore cavity and thus bringing the metal–organic layers closer to each other. The distorted-octahedral geometry of the (MOF_5_)^2–^ pillars (where M = V^5+^, Nb^5+^, or Ta^5+^), which displaces the metal cation from the center of the octahedron and closer to the coordinating oxygen atom, has furthermore been found to influence the tilting of the pyz-rings (δ_pyz,VOFFIVE-1-Ni_ = 29.7°, δ_pyz,NbOFFIVE-1-Ni_ = 30.1°, and δ_pyz,TaOFFIVE-1-Ni_ = 37.1° from the *c*-axis) as well as the rotation of the anionic unit (δ_(VOF5)_ = 11.6°, δ_(NbOF5)_ = 34.1°, and δ_(TaOF5)_ = 27.1° between adjacent pillars) (Figure 1b–f and Figures S7–S12).^18,22^ The tilting and rotational freedom of these building units may become more limited as the distance between the pyz-ligand and pillaring units shorten. This limitation can significantly influence the shape and size of the pore cavity (Figure 1b–g) and plays an important role in controlling the accessibility as well as the diffusion of guest molecules.^18^ The pore sizes in the as-synthesized NbOFFVIE-1-Ni and TaOFFIVE-1-Ni were found to be quite comparable (Figure 1c,d and Figure 1f,g) due to the similar physiochemical properties of the Nb^5+^ and Ta^5+^ cations. In comparison, the comparatively small ionic radius and high electronegativity of the V^5+^ cation was found to shorten the bond length between the coordinating atoms in the anionic pillar (*d*_V–Feq_ = 1.883 Å, *d*_V__–F/O_ = 1.858 Å for VOFFIVE-1-Ni compared to *d*_V–Feq_ = 1.894 Å, *d*_V–F/O_ = 1.981 Å and *d*_V–Feq_ = 1.893 Å, *d*_V–F/O_ = 2.029 Å for NbOFFIVE-1-Ni and TaOFFIVE-1-Ni, respectively) (Figures S7, S9, and S11), leading to a slight reduction of the effective pore aperture (Figure 1 and Figures S7–S12). Based on these observations, VOFFIVE-1-Ni can therefore be assumed to be more structurally rigid compared to NbOFFIVE-1- Ni and TaOFFIVE-1-Ni. In contrast, the substitution of the pyz-ligand with pyz-NH_2_ resulted in a significant reduction of the pore dimensions and a subsequent increased framework density. The accessibility of guest molecules into the pore structure may therefore be severely limited, and further details (e.g., crystal structure, sorption properties) regarding the HUM can be found in the Supporting Information. The thermal and hydrolytic stabilities of the as-synthesized HUMs (Figure S21 and S22) were furthermore found to be comparable to those of NbOFFIVE-1-Ni due to similarities in the nucleophilicity of the (MOF_5_)^2–^-pillars in the materials. The HUMs were also observed to be stable at temperatures up to 573 K and retained their crystallinity after exposure to water for 3 months.

**Figure 1:**
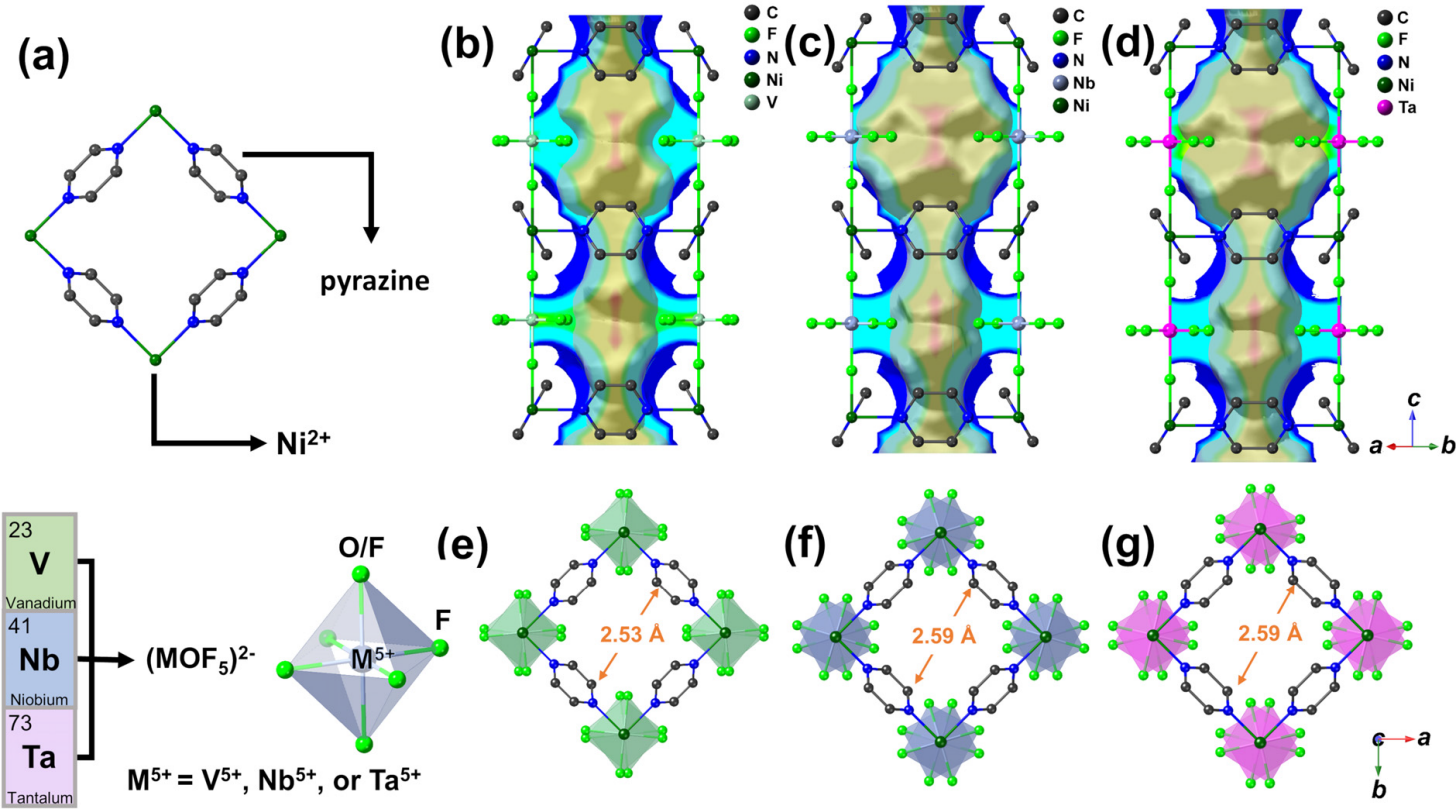
(a) Building blocks of MOFFIVE-1-Ni including the 2D metal–organic (Ni(pyz)_2_) layer as well as the modifiable anionic pillar (MOF_5_)^2–^ (where M = V^5+^, Nb^5+^, or Ta^5+^). Images of the crystal structure superimposed onto a cross-section of a distance map showing the pore structure (generated by taking the van der Waals radii of the atoms into account) and the solvent-excluded surface (obtained using a probe radius of 1.2 Å) contoured in gray for (b) VOFFIVE-1-Ni·2H_2_O, (c) NbOFFIVE-1-Ni·CH_3_OH, (d) TaOFFIVE-1-Ni·CH_3_OH (solvent molecules were excluded for clarity). The crystal structure viewed along [001] showing the rotational displacement of the anionic pillar between two adjacent layers as well as the pyz- and pillar-limiting pore apertures in (e) VOFFIVE-1-Ni·2H_2_O, (f) NbOFFIVE-1-Ni·CH_3_OH, and (g) TaOFFIVE-1-Ni·CH_3_OH.

The size and physiochemical properties of the anionic pillar in the HUMs are pivotal in controlling the pore dimensions and accessibility of guest molecules to the structures. However, the dynamical movement of the pyz-ligand and pillaring units, although limited, has been observed to enable the adsorption of molecules larger than the aperture-limiting pore size. This has been shown by the adsorption of C_3_H_6_ (4.68 Å)^19^ in NbOFFIVE-1-Ni (3.047 Å pore size).^18^ The accessibility of guest molecules is therefore also dependent on the rigidity of the HUM structure, in addition to the size of the sorbent molecule and sorbent–sorbate interactions. According to N_2_ sorption data recorded at 77 K the Brunauer–Emmett–Teller (BET) specific surface areas of the HUMs were 26, 142, and 190 m^2^ g^–1^ for VOFFIVE-1-Ni, TaOFFIVE-1Ni, and NbOFFIVE-1-Ni, respectively. The trend in BET surface area appears to agree well with the observed structural properties of the HUMs—VOFFIVE-1-Ni exhibits reduced dynamical movement of the pyz-ligand and (VOF_5_)^2–^-pillar at cryogenic temperatures, which in turn significantly limits the accessibility of N_2_ molecules into the framework, in contrast to NbOFFIVE-1-Ni and TaOFFIVE-1-Ni. The sorption properties of the HUMs were further probed by using CO_2_, CH_4_, and N_2_ isotherms recorded at 293 K (Figure 2b,c). Preferential adsorption of CO_2_ was observed in the HUMs as compared to CH_4_ and N_2_—VOFFIVE-1-Ni (13.18 wt %, 3.00 mmol g^–1^) was found to have the highest gravimetric CO_2_ uptake capacity at 1 bar followed by NbOFFIVE-1-Ni (10.70 wt %, 2.43 mmol g^–1^) and TaOFFIVE-1-Ni (9.48 wt %, 2.15 mmol g^–1^). The low-pressure-range (<0.1 bar) CO_2_ uptake of TaOFFIVE-1-Ni and VOFFIVE-1-Ni (Figure 2c) was slightly lower compared to that of NbOFFIVE-1-Ni, which shows that NbOFFIVE-1-Ni may possess pores with more suitable dimensions than TaOFFIVE-1-Ni and VOFFIVE-1-Ni for CO_2_ capture at low concentrations. The shape of the CO_2_ adsorption isotherms in this pressure range, furthermore, indicates that the interactions between the CO_2_ molecules and pore surface of the HUMs are similar for NbOFFIVE-1-Ni and TaOFFIVE-1-Ni. The adsorption isotherm for VOFFIVE-1-Ni, on the other hand, shows an inflection point at approximately 1.3 mbar. A similar response has been seen in the adsorption isotherms of xenon (Xe) in SIFSIX-3-Ni^23^ and CH_4_ in ZU-66 (composed of Zn^2+^, 4,4′-bipyridineacetylene ligands, and (ZrF_6_)^2–^-pillars).^24^ The inflection point in the Xe adsorption isotherm was attributed to the rotational rearrangement of the pyz-ligands from a disordered tilting arrangement to an ordered arrangement upon Xe loading, which occurred in order to enhance guest–host interactions.^23^ Similar configurational ordering of the pyz-ligand and/or anionic pillar may take place in VOFFIVE-1-Ni during the adsorption of CO_2_, which could explain the presence of the inflection point in the isotherm as well as the increased CO_2_ sorption capacity at higher pressure (>0.1 bar). At 1 bar, the uptake of N_2_ and CH_4_ on these HUMs was found to be moderately low in contrast to the CO_2_ uptake (Figure 2b). In particular, VOFFIVE-1-Ni has the highest N_2_ (1.23 wt %, 0.44 mmol g^–1^) and the lowest CH_4_ (0.07 wt %, 0.04 mmol g^–1^) uptake, which may indicate a possible expansion of the pore aperture size in VOFFIVE-1-Ni at room temperature, leaving the structure slightly accessible to N_2_ (3.6 Å) while omitting the entry of CH_4_ (3.8 Å). The effective sieving of CO_2_ from CH_4_ is a highly desirable property, especially for applications such as biogas upgrading in which separation of these components is required in gas mixtures typically composed of 50% v/v CO_2_ and 50% v/v CH_4_.^25^ The CO_2_ and CH_4_ uptake at 0.5 bar (Figure 2e) shows that VOFFIVE-1-Ni has a high uptake for CO_2_ (12.06 wt %, 2.75 mmol g^–1^) and low CH_4_ uptake (0.002 wt %, 1.2 μmol g^–1^) as compared to NbOFFIVE-1-Ni (10.28 wt %, 2.33 mmol g^–1^ CO_2_ and 0.13 wt %, 0.08 mmol g^–1^ CH_4_) and TaOFFIVE-1-Ni (9.03 wt %, 2.05 mmol g^–1^ CO_2_ and 0.06 wt %, 0.04 mmol g^–1^ CH_4_). Furthermore, comparing the CO_2_/CH_4_ (50:50) uptake ratio of the HUMs with those of other MOM sorbents (Figure 2f) shows that VOFFIVE-1-Ni possesses a far greater uptake ratio (2280) as compared to other materials such as UTSA-280 (30),^26^ Cu–F pymo (48),^27^ Qc-5-Cu-dia (72),^28^ and [Cd_2_L(H_2_O)_2_·5H_2_O] (73)^29^ (Figure 2f). The water uptake of the HUMs was evaluated at 293 K (Figure 2d) and showed that all HUMs had low H_2_O uptake at *p*/*p*° = 1 which ranged from 0.11 to 0.15 g g^–1^ (6.07–8.29 mmol g^–1^). The somewhat hydrophobic nature of the HUMs is highly advantageous especially when capture and separation occur in gas mixtures saturated with water vapor, which is the case for raw biogas.^25^ The lack of hysteresis in the sorption isotherm further affirms the structural stability of the materials in the presence of water vapor, which is in agreement with the recorded PXRD patterns of the H_2_O-soaked HUMs (Figure S22).

**Figure 2:**
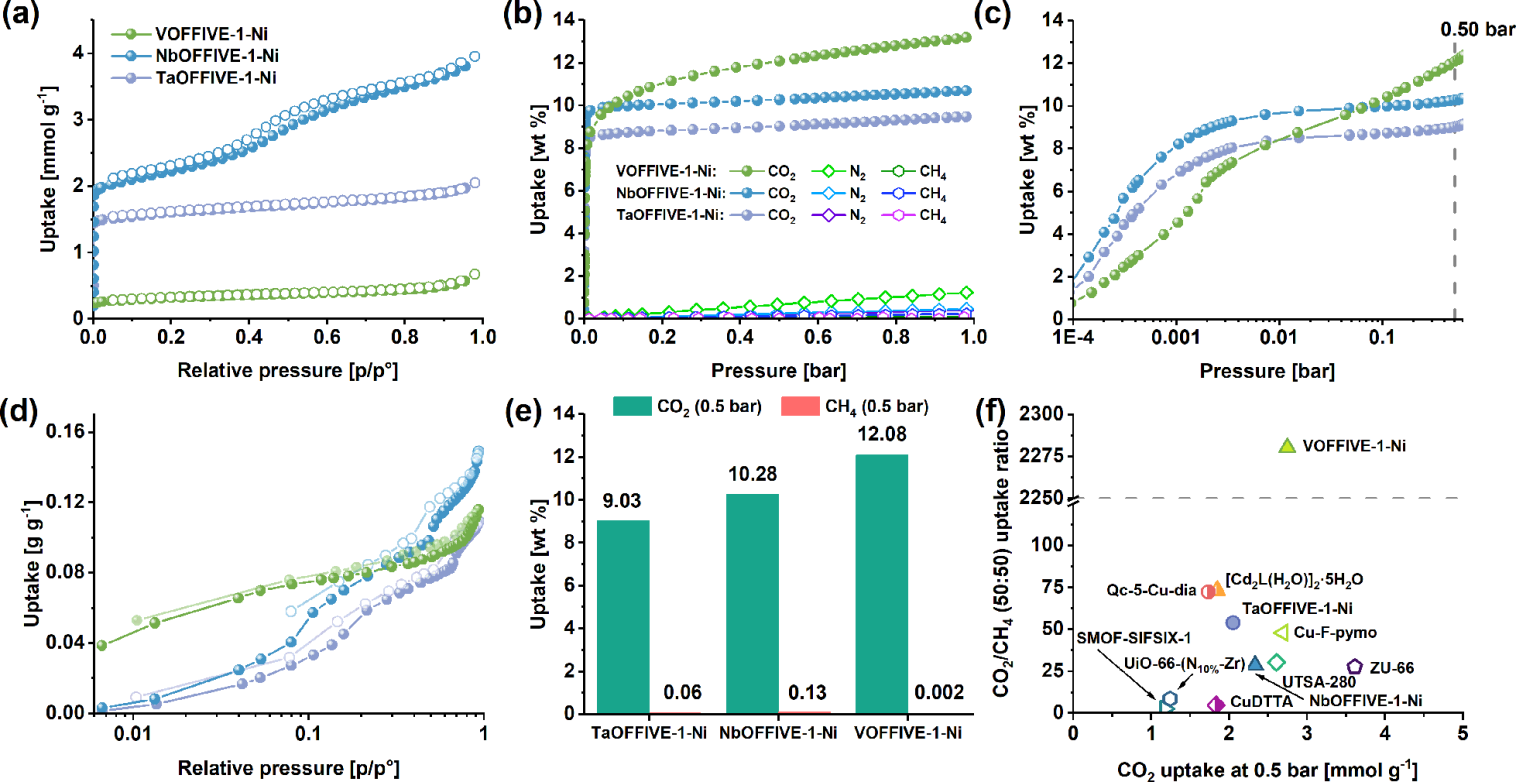
(a) N_2_ sorption isotherms recorded at 77 K, (b) CO_2_, CH_4_, and N_2_ adsorption isotherms recorded at 293 K, (c) the low-pressure region of the CO_2_ adsorption isotherms, (d) H_2_O sorption isotherms recorded at 293 K, (e) comparison of CO_2_ and CH_4_ uptakes at 0.5 bar, 293 K, and (f) comparison of the CO_2_/CH_4_ (50:50) uptake ratio and CO_2_ uptake capacity at 0.5 bar for the synthesized HUMs as well as other porous framework materials.^26−33^ Filled and hollow circles in the sorption isotherms represent the adsorption and desorption branches, respectively.

Time-dependent *in situ* infrared spectroscopy (IR) was used to further investigate the interaction between the CO_2_ molecules and the HUMs (Figure 3 and Figures S33–S36). A perturbation of the ν(CH) (approximately 3000–3030 cm^–1^) and δ(CH)_as_+ν(CC)_as_ (approximately 1390–1430 cm^–1^) bands corresponding to the pyz-ligand^10,34^ was seen upon CO_2_ adsorption (at a partial pressure of 0.13 kPa) in the HUMs (Figure 3a–c). Significant blue shifts of these bands were observed for NbOFFIVE-1-Ni and TaOFFIVE-1-Ni, while a major perturbation was only seen for the δ(CH)_as_+ν(CC)_as_ band in VOFFIVE-1-Ni. Based on the IR spectra, these distinct differences likely indicate a reorientation of the pyz-ligands in NbOFFIVE-1-Ni and TaOFFIVE-1-Ni. Fourier difference data have previously indicated that the adsorbed CO_2_ molecules occupy an energetically favorable position within the channels of NbOFFIVE-1-Ni^17^ that places the electronegative oxygen atoms in close proximity to the hydrogen atoms of the pyz-ligand, which in turn can influence the orientation of the ligands. The small perturbation of the ν(CH) bands in VOFFIVE-1-Ni, on the other hand, suggests that the pyz-ligands are in a different configuration and that the structure of VOFFIVE-1-Ni is affected in a distinctly different manner by the adsorbed CO_2_ molecules. This assumption is supported by the appearance of the δ(CH)_as_+ν(CC)_as_ band that is blue-shifted to slightly higher vibrational frequencies compared to NbOFFIVE-1-Ni and TaOFFIVE-1-Ni. The ν_3_(CO_2_) region of the IR spectra was further evaluated in order to study the interaction between the CO_2_ molecules and the HUM frameworks in more detail (Figure 3d–f). The emergence of an asymmetric stretching band of CO_2_ (ν_3_(^12^CO_2_)_as_) was seen in all samples after 0.3 s followed by a weak band at approximately 2271–2273 cm^–1^ corresponding to ν_3_(^13^CO_2_).^35,36^ The intensity of the ν_3_(^12^CO_2_)_as_ band increased rapidly within the first minutes of adsorption on VOFFIVE-1-Ni followed by NbOFFIVE-1-Ni and TaOFFIVE-1-Ni. VOFFIVE-1-Ni was observed to interact more strongly with the CO_2_ molecules compared to NbOFFIVE-1-Ni and TaOFFIVE-1-Ni, which can be seen from the position of the ν_3_(CO_2_)_as_ band at 2337 cm^–1^ for VOFFIVE-1-Ni, as compared to 2343 cm^–1^ for NbOFFIVE-1-Ni and 2345 cm^–1^ for TaOFFIVE-1-Ni. The presence of combination bands corresponding to (ν_3_+ν_1_)CO_2_ and (ν_3_+2ν_2_)CO_2_ at approximately 3695 and 3594 cm^–1^ were also seen on both VOFFIVE-1-Ni and NbOFFIVE-1-Ni^37^ as well as a third combination “hot band”, ((ν_3_+ν_2_)(CO_2_)-ν_2_(CO_2_)), at 2326 and 2328 cm^–1^, respectively, which are characteristic for adsorbed CO_2_ species.^35,38,39^ The appearance of a single ν_3_(CO_2_)_as_ band furthermore points to the presence of a single adsorption site for CO_2_ in the HUMs, which is in good agreement with previously published experimental results as well as theoretical calculations on NbOFFIVE-1-Ni^17^ and SIFSIX-3 HUMs.^10,40^

**Figure 3:**
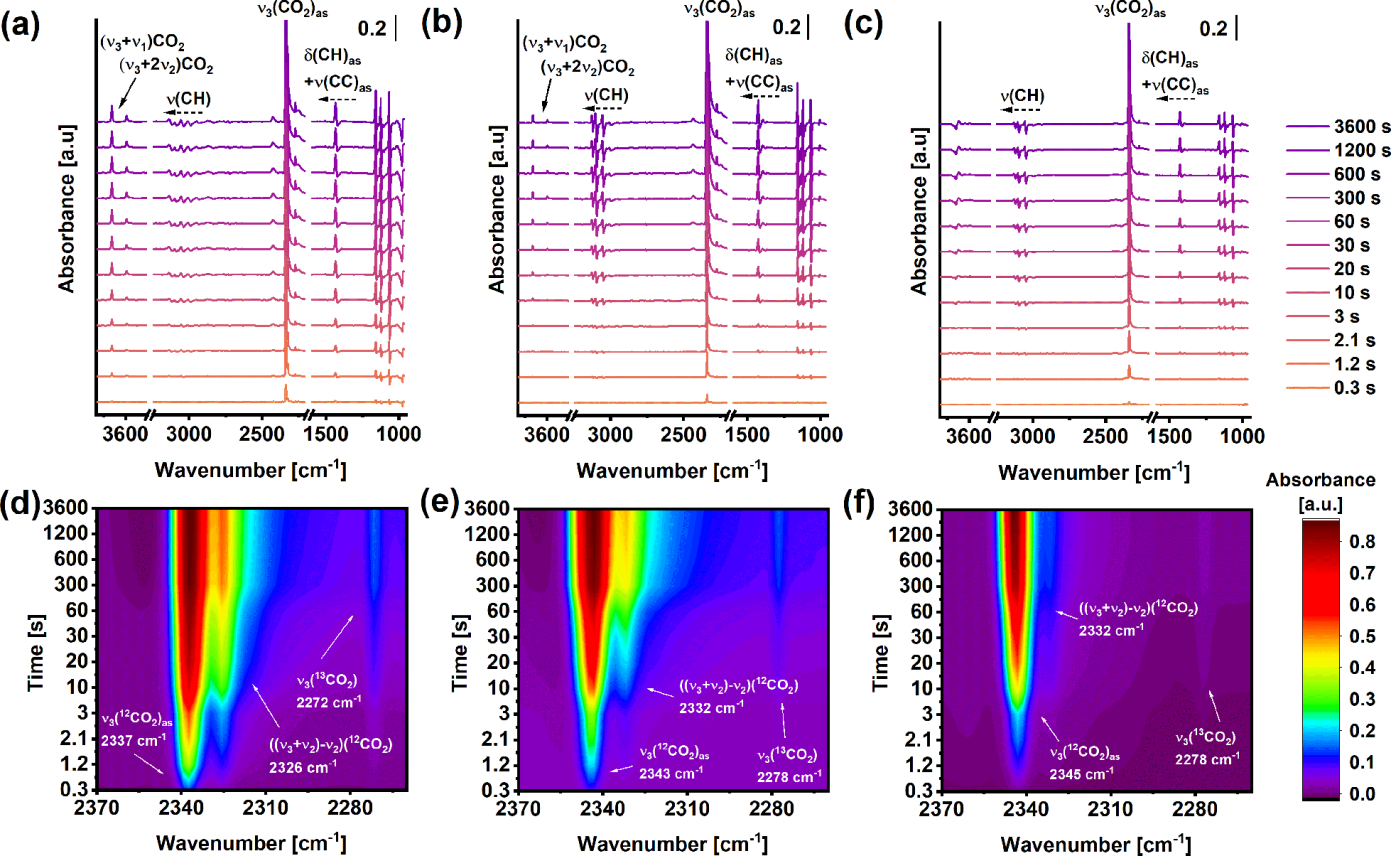
Time-dependent *in situ* IR spectra showing CO_2_ adsorption on (a) VOFFIVE-1-Ni, (b) NbOFFIVE-1Ni, and (c) TaOFFIVE-1-Ni and contour maps of the ν_3_(CO_2_) region of (d) VOFFIVE-1-Ni, (e) NbOFFIVE-1-Ni, and (f) TaOFFIVE-1-Ni.

Differences in the guest–host interactions between VOFFIVE-1-Ni and NbOFFIVE-1-Ni as well as between TaOFFIVE-1-Ni were further evaluated from their isosteric enthalpies of CO_2_ adsorption (Δ*H*_ads_) (Figure 4a). VOFFIVE-1-Ni was found to have the lowest −Δ*H*_ads_ ranging from 57 to 66 kJ mol^–1^ (at 0.38–0.82 mmol g^–1^ CO_2_ loading) compared to NbOFFIVE-1-Ni (48–53 kJ mol^–1^ at 0.82–1.68 mmol g^–1^ CO_2_ loading) and TaOFFIVE-1-Ni (45–49 kJ mol^–1^ at 0.64–1.48 mmol g^–1^ CO_2_ loading). These results correlate well with the observed frequency difference of the ν_3_(CO_2_) band in the *in situ* IR spectra (Figure 3c,d) for the different samples as well as the higher degassing temperature required to regenerate VOFFIVE-1-Ni (Figure S24). Moreover, they also give credence to the assumption that VOFFIVE-1-Ni may possess energetically favorable adsorption sites that can form strong intermolecular interactions with CO_2_ molecules. Furthermore, the CO_2_ adsorption rate (Figure 4b), obtained from the ν_3_(CO_2_)_as_ bands, was found to be rapid in all HUMs, in particular VOFFIVE-1-Ni. The adsorption process was observed to reach equilibrium after 114 s, with the fastest CO_2_ adsorption occurring in VOFFIVE-1-Ni (90% uptake reached within 20 s) followed by NbOFFIVE-1-Ni (97 s) and TaOFFIVE-1-Ni (114 s). The calculated diffusion time constants (*Dr*^*–2*^) (Figure S38) show that VOFFIVE-1-Ni (4.8 × 10^–2^ s^–1^) exhibits a 12 and 6 times faster CO_2_ diffusion compared to NbOFFIVE-1-Ni (4.1 × 10^–3^ s^–1^) and TaOFFIVE-1-Ni (7.8 × 10^–3^ s^–1^), respectively. The large difference in adsorption rate may, as previously mentioned, be attributed to a structural reorientation of the pyz-ligands in VOFFIVE-1-Ni that may increase the accessibility of CO_2_ molecules in the framework and lead to an increase in the guest molecules’ diffusivity.

**Figure 4:**
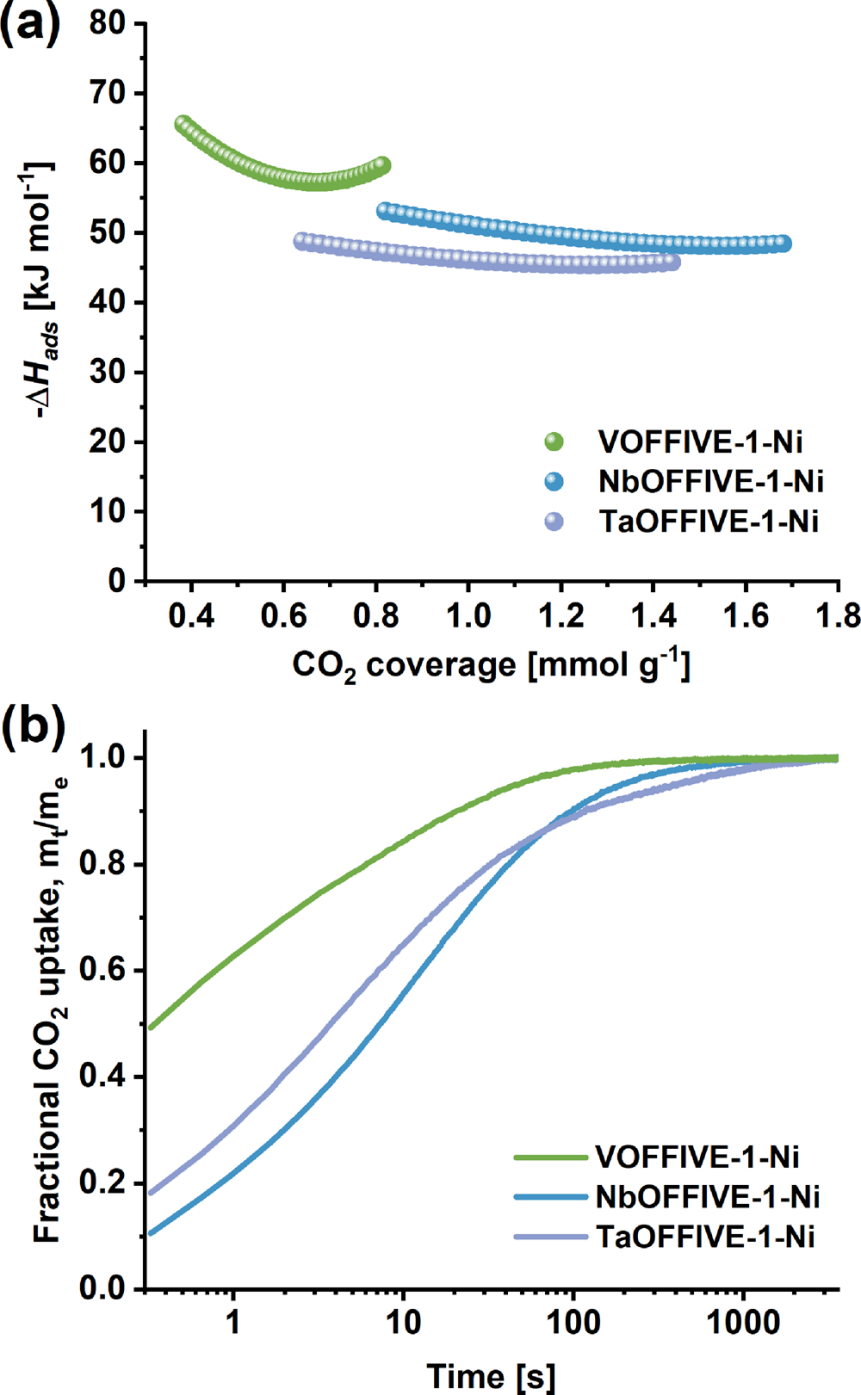
(a) Isosteric enthalpies of CO_2_ adsorption and (b) CO_2_ adsorption profiles derived from the ν_3_(^12^CO_2_) band obtained from the *in situ* IR spectra of the pyz-based HUMs.

We demonstrated how pillar substitution in NbOFFIVE-1-Ni can be used to tune the sorption and molecular sieving properties of the isoreticular HUM materials. In particular, VOFFIVE-1-Ni is shown to possess an improved gravimetric CO_2_ uptake capacity at high pressures (i.e., >0.1 bar) compared to NbOFFIVE-1-Ni as well as excellent molecular sieving of CH_4_ from CO_2_, as indicated by the material’s high CO_2_/CH_4_ (50:50) uptake ratio above 2250. Future studies, including breakthrough analysis, would enable the further optimization of VOFFIVE-1-Ni for application. The material appears to exhibit a unique structural response likely owing to a controlled but limited rotational freedom of the pyz-ligand and anionic pillaring unit in the structure. The strong binding sites for CO_2_ result in fast CO_2_ adsorption kinetics as well as low enthalpies (−Δ*H*_ads_) of CO_2_ adsorption. In conjunction, the excellent thermal and hydrolytic stability of VOFFIVE-1-Ni makes it a highly promising sorbent for CO_2_ capture and separation from CH_4_ and applications related to biogas upgrading.

## Supporting Information Available

Details regarding the synthesis of HUM materials as well as characterization, including structure solution using 3D ED, Rietveld and Pawley refinements, stability studies, porosity and surface areas, CO_2_, N_2_, and CH_4_ sorption isotherms, and calculations pertaining to CO_2_ enthalpies of adsorption, *in situ* infrared spectra, and scanning electron microscopy images (PDF)
