# Supplementary material for: Achieving Molecular Sieving of CO2 from CH4 by Controlled Dynamical Movement and Host–Guest Interactions in Ultramicroporous VOFFIVE-1-Ni by Pillar Substitution
Source: Nano Lett. 2024 May 30;24(25):7616–22. doi: 10.1021/acs.nanolett.4c01305 (PMC11212043; doi:10.1021/acs.nanolett.4c01305)
Supplement: Supplementary file 1 — nl4c01305_si_001.pdf [file nl4c01305_si_001.pdf]

Achieving Molecular Sieving of CO_2 from CH_4 by Controlled Dynamical Movement and Host-Guest Interactions in Ultramicroporous VOFFIVE-1-Ni by Pillar Substitution

Supporting Information

Ribooga Chang,[†] Zoltán Bacsik,[‡] Guojun Zhou,[‡] Maria Strømme,[†] Zhehao Haung,[‡] Michelle Åhlén,[†,*] and Ocean Cheung,[†,*]

†Division of Nanotechnology and Functional Materials, Department of Materials Science and Engineering, Ångström Laboratory, Uppsala University, Box 35 SE-751 03, Uppsala, Sweden.

‡Department of Materials and Environmental Chemistry, Stockholm University, SE-106 91, Stockholm, Sweden.

*Email for M.Å.: michelle.ahlen@angstrom.uu.se

*Email O.C.: ocean.cheung@angstrom.uu.se

Table of contents

S1. Materials........S-2

S2. Experimental section........S-2

S2.1. Synthesis of [Ni(VOF_5)(pyrazine)$_2$·2H_2O] (VOFFIVE-1-Ni·2H_2O), [Ni($NbOF_5$)(pyrazine)$_2$·CH_3OH] (NbOFFIVE-1-Ni·CH_3OH), and [Ni($TaOF_5$)(pyrazine)$_2$·CH_3OH] (TaOFFIVE-1-Ni·CH_3OH)........S-2

S2.2. Synthesis of [Ni($NbOF_5$)(2-aminopyrazine)$_2$] (NbOFFIVE-2-Ni)........S-2

S3.1. Structure solution using 3D electron diffraction........S-3

S.3.1.1. Transmission electron microscopic (TEM) analysis........S-3

S.3.1.2. Continuous rotation electron diffraction (cRED) collection........S-3

S.3.1.3. cRED data processing........S-4

S3.2. Rietveld and Pawley refinements........S-9

S3.2.1. Rietveld refinement........S-9

S3.2.2. Pawley refinement........S-21

S3.3. Stability study........S-26

S3.3.1. Thermal decomposition........S-26

S3.3.2. Hydrolytic stability........S-27

S3.4. Gravimetric CO_2 adsorption cycles and sorbent regeneration........S-28

S3.5. Surface area and porosity........S-29

S3.5.1. CO_2, N_2, and CH_4 sorption isotherms........S-33

S3.5.2. Isosteric enthalpies of CO_2 adsorption........S-33

S3.5.3. H_2O sorption isotherm of NbOFFIVE-2-Ni........S-36

S3.6. *In situ* infrared spectroscopy........S-36

S3.7. Scanning electron microscopy images........S-40

S3.8. CO_2 adsorption kinetics........S-40

S4. References........S-42

S1. Materials

Tantalum(V) oxide: Ta_2O_5 (99% purity, Thermo Scientific), Vanadium(V) oxide: V_2O_5 (> 99% purity, Thermo Scientific), Niobium(V) oxide: Nb_2O_5 (99.99% purity, Thermo Scientific), Hydrofluoric acid: HF (48%, Sigma-Aldrich), Nickel nitrate hexahydrate: $Ni(NO_3)_2{\cdot}6H_2O$ (99 < x < 102%, Sigma-Aldrich), Pyrazine (≥ 99% purity, Sigma-Aldrich), Aminopyrazine (98% purity, Sigma-Aldrich) Methanol (≥ 99.9% purity, Sigma-Aldrich). All chemicals were used without further purification.

S2. Experimental section

S2.1. Synthesis of [Ni(VOF_5)(pyrazine)$_2{\cdot}2H_2O$] (VOFFIVE-1-Ni·$2H_2O$), [Ni($NbOF_5$)(pyrazine)$_2{\cdot}CH_3OH$] (NbOFFIVE-1-Ni·CH_3OH), and [Ni($TaOF_5$)(pyrazine)$_2{\cdot}CH_3OH$] (TaOFFIVE-1-Ni·CH_3OH)

The synthesis of the pyz-based HUMs was carried out in a similar manner to procedures for NbOFFIVE-1-Ni[1] and TaOFFIVE-1-Ni[2], with slight modifications. Briefly, 0.3 mmol of V_2O_5 (54.6 mg), Nb_2O_5 (79.7 mg), or Ta_2O_5 (132.6 mg) was mixed with 0.4 ml of HF in a 25 ml Teflon vessel. The mixture was sealed in a stainless-steel autoclave and heated at 403 K (130 °C) overnight. After cooling down the pre-heated mixture to room temperature, 3 ml of deionized water, 4.8 mmol of pyrazine (384.4 mg), and 0.6 mmol of $Ni(NO_3)_2{\cdot}6H_2O$ (174.5 mg) were added into the mixture, and thereafter left on an orbital shaker at 250 ppm for 3 h. After mixing, the mixture was sealed and reacted at 403 K (130 °C) for NbOFFIVE-1-Ni and TaOFFIVE-1-Ni, and 358 K (85 °C) for VOFFIVE-1-Ni, respectively, for 48 h. After the reaction, pastel light-blue (TaOFFIVE-1-Ni), light-violet (NbOFFIVE-1-Ni), and light-emerald (VOFFIVE-1-Ni) microcrystalline powers were obtained. The powders were collected by centrifugation at 3,800 rpm for 10 min and washed twice using methanol. After washing, samples were soaked in methanol and solvent exchange for 24 h. The soaked powders were thereafter centrifuged and then dried at 343 K (70 °C) for 24 h. While some variation was observed between sample batches, each of the three materials yielded an average of 200 mg of powder (corresponding to 75%, 73%, and 61% based on V, Nb, and Ta, respectively).

S2.2. Synthesis of [Ni($NbOF_5$)(2-aminopyrazine)$_2$] (NbOFFIVE-2-Ni)

In order to synthesize NbOFFIVE-2-Ni a mixture of 0.3 mmol of Nb_2O_5 (79.7 mg) in 0.4 ml of HF was heated at 403 K (130 °C) overnight. After cooling, 3 ml of deionized water, 0.6 mmol of $Ni(NO_3)_2{\cdot}6H_2O$ (174.5 mg), and 4.8 mmol of 2-aminopyrazine (456.5 mg) was added into the pre-heated mixture and mixed on a shaker at 250 rpm for 3 h. After mixing, the mixture was sealed and heated at 403 K (130 °C) for 36 h. After the reaction, dark-brown (NbOFFIVE-2-Ni) powder was obtained and carried out the same washing, solvent-exchange, and drying steps. As a final product, an average yield of 150 mg of powder was obtained (corresponding to 50% based on Nb).

S3. Characterization

Powder X-ray diffraction patterns of all synthesized HUMs were collected on a Bruker D8 powder diffractometer (Karlsruhe, Germany) using Cu-K$\alpha_{1,2}$ radiation (λ_1 = 1.5406 Å, λ_2 = 1.5444 Å) and operated at 40 kV and 40 mA. A capillary holder was employed for all measurements and patterns were collected in a 2θ-range of 5 to 100° using a step size of 0.01° at ambient conditions. Scanning electron microscopy (SEM) images were taken using a Zeiss Merlin Field Emission Scanning Electron Microscope (Oberkochen, Germany) using an acceleration voltage of 2 kV. Volumetric CO_2, N_2, and H_2O sorption isotherms were recorded on HUM samples activated at 423 K for 3 h under dynamic vacuum (1×10^{-4} Pa) using a Micromeritics SmartVacPrep (Norcross, USA). After activation, equilibrium N_2 sorption isotherms were recorded at 77 K as well as CO_2, N_2, and H_2O sorption isotherms at 293 K which were collected using a temperature-controlled water bath at 293 K on a Micromeritics ASAP2020 surface area analyzer (Norcross, USA). Heat of adsorption was calculated using Clausius-Clapeyron equation (Equation 7) on the CO_2 adsorption recorded at 423 K and 433 K. CO_2/CH_4 selectivity in this study is reported as the uptake ratio between CO_2 and CH_4 and the use of ideal adsorption solution theory (IAST) was omitted due to that molecular sieving nature of the samples, which falls outside one of assumptions of IAST where each sorbent should have equal accessibility to the sorbate area. Thermogravimetric profiles were recorded on a Mettler Toledo Thermal Analysis System TGA 2 and Mettler Toledo Thermal Analysis System TGA/DSC 3+ (Columbus, United States) using air, N_2, and/or CO_2 at a flow-rate of 50 ml min^{-1}.

S3.1. Structure solution using 3D electron diffraction

S.3.1.1. Transmission electron microscopic (TEM) analysis

Specimens were loaded onto copper grids coated with a layer of carbon. The investigative procedures were conducted using a JEOL JEM2100 microscope (Akishima, Japan) operating at 200 kV. The TEM images were recorded utilizing a Gatan Orius 833 CCD camera with a resolution of 2048 x 2048 pixels and an individual pixel dimension of 7.4 µm. Furthermore, the collection of electron diffraction patterns was facilitated by a Timepix pixel detector QTPX-262k, featuring a pixel count of 512 x 512 and a pixel size of 55 µm. This pixel detector was innovatively manufactured by Amsterdam Scientific Instruments (Amsterdam, Netherlands).

S.3.1.2. Continuous rotation electron diffraction (cRED) collection

The data acquisition was carried out employing *Instamatic* software,[3] utilizing a single-tilt tomography holder with a range of −70° to +70° for tilting within the TEM. The cRED data collection involved the use of an approximately 0.6 µm diameter aperture. Goniometer tilt occurred at a speed of 1.1° s^{-1}, and each frame was exposed for 0.3 s. This strategic approach ensured that each data set was assembled

within a span of 2 min, thereby minimizing beam-related degradation and enhancing data quality. The integration of data sets for structure determination harnessed the capabilities of XDS packages.[4]

S.3.1.3. cRED data processing

Due to the small crystal size of NbOFFIVE-2-Ni (< 2 μm), 3DED, namely continuous rotation electron diffraction (cRED),[5, 6] was applied for crystal structure analysis. Reconstructed 3D reciprocal lattice from the cRED data indicated the unit cell parameters to be a = 10.13 Å, b = 10.45 Å, c = 8.21 Å, α = 90.01°, β = 89.90°, γ = 90.03°. NbOFFIVE-2-Ni furthermore crystallized in tetragonal system. The two-dimensional (2D) sliced cuts of the 3D reciprocal lattice exhibited the reflection conditions *hk*0: $h + k = 2n$, 0*k*0: k = 2n (Figure S1). Based on these reflection conditions, the space group was found to be *P*4/*nbm* (No. 125) and it was used to conduct structure determination for NbOFFIVE-2-Ni.

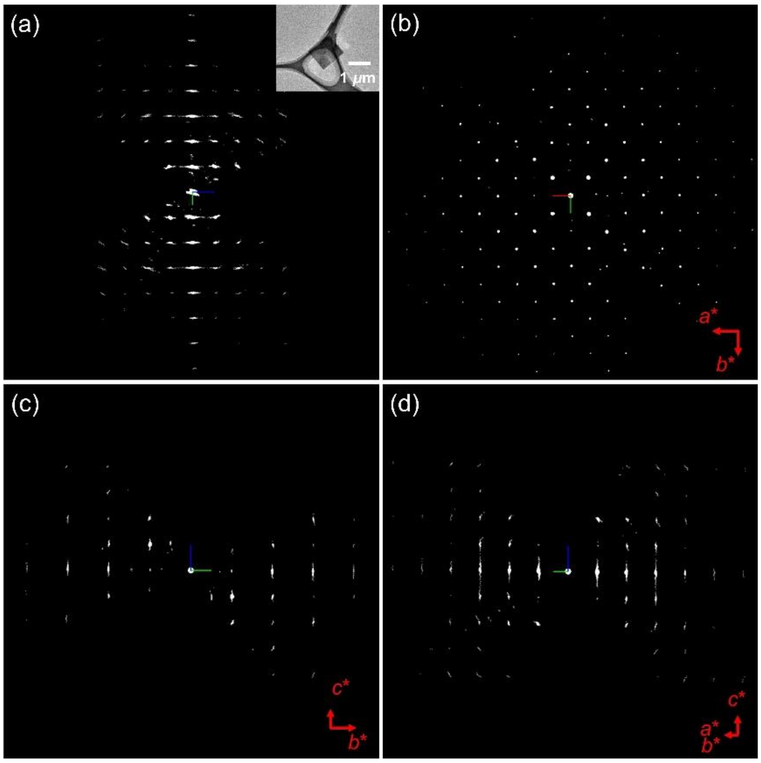

Figure S1. a) Reconstructed 3D reciprocal lattice of NbOFFIVE-2-Ni (inset is the image of the crystal on which the cRED data was collected) and 2D slice cuts of 3D reciprocal lattice of NbOFFIVE-2-Ni show the (b) *hk*0, (c) 0*kl*, and (d) *hkl* planes.

The dataset exhibited a high resolution of 0.80 Å with data completeness 86.0% for NbOFFIVE-2-Ni.[7, 8] Due to the high 3D electron diffraction data quality, the structure could be solved via ab initio by direct methods. The final refinement converged to a R_1 value of 0.1408. Details of data collection and structure refinement are shown in Table S1.

The refinement of NbOFFIVE-2-Ni's unit cell parameters (Figure S2) was carried out through Pawley fitting by using TOPAS 4.1. Background correction was performed using a 16th-order Chebychev

polynomial. Refinement was executed utilizing a Pearson VII-type peak profile function, followed by the refinement of unit cell and zero-shift parameters. Finally, the unit cell parameters were refined to $a = b = 9.901(5)$ Å and $c = 8.355(8)$ Å. The corresponding R-values were converged to: $R_p = 0.0433$, $R_{wp} = 0.0582$, and $R_{exp} = 0.0514$ (Table S2). Unit cell parameters obtained from cRED data are typically larger than those obtained from X-ray diffraction. This is generally assumed to be due to lens distortions and changes in sample height during data collection.[9]

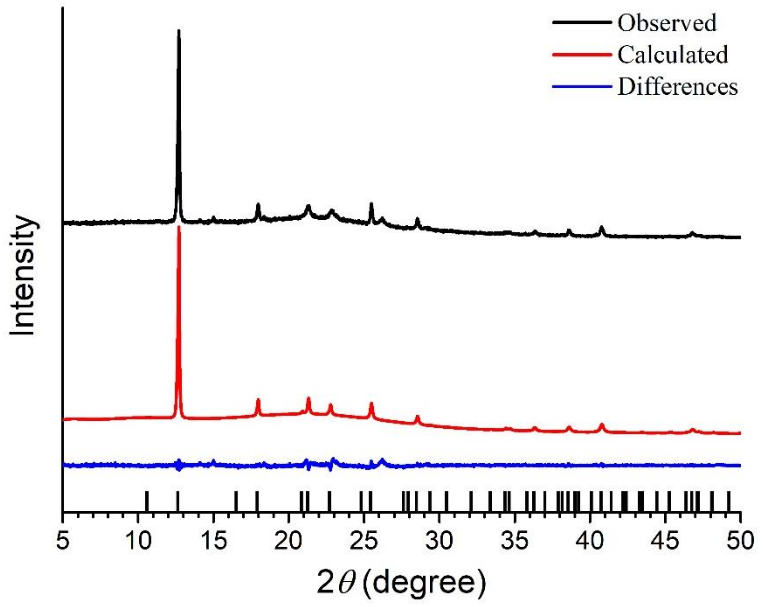

Figure S2. Pawley fitting against powder X-ray diffraction (λ = 1.5406Å) for NbOFFIVE-2-Ni. Red line: calculated; black line: observed; blue line: difference; black bars: Bragg conditions.

To further validate the structure of NbOFFIVE-2-Ni, the PXRD pattern was simulated by using the structural model obtained from single crystal analysis and unit cell parameters refined by Pawley fitting, and it matched well with the observed pattern (Figure S3).

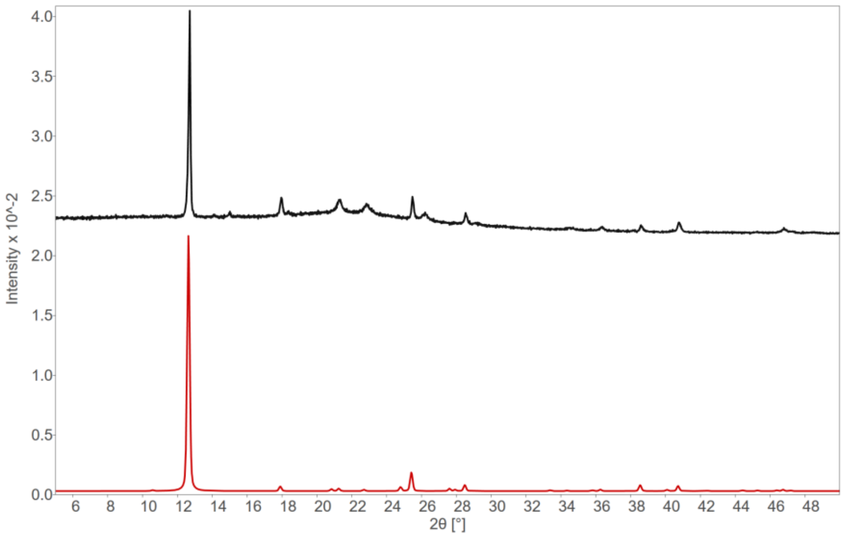

Figure S3. Simulated PXRD pattern (λ = 1.5406 Å) of NbOFFIVE-2-Ni by using the structural model obtained from single crystal analysis.

Table S1. Single crystal data collection and crystallographic data for NbOFFIVE-2-Ni (λ = 0.0251 Å).

Sample	NbOFFIVE-2-Ni
Tilt range [°]	−39.05° to 73.86°
Tilt rate [° s^{-1}]	1.1
Exposure time [s]	0.3
Total number of images	250
Data collection time [min]	1.6
Beam current	< 0.01 pA

Chemical formula	$CF_{0.75}N_{0.75}Nd_{0.06}Ni_{0.19}$
Formula weight	56.57
Crystal system	Tetragonal
Space group	*P*4/nbm (No. 125)
a [Å]	10.3400(15)
b [Å]	10.3400(15)
c [Å]	8.1300(16)
α [°]	90
β [°]	90
γ [°]	90
Z	16
Cell volume [$Å^3$]	869.2(3)
Completeness [%]	86.0 %
Resolution [Å]	0.8
No. of reflections (all unique)	420
No. of reflections ($F_o>4\sigma(F_o)$)	3536
Refined parameters	33
R_{int}	0.1556
R_1 ($F_o>2\sigma(F_o)$)	0.1408
R_1 (all reflections)	0.1639
Goof	1.289

Table S2. Crystallographic details of Pawley fitting of NbOFFIVE-2-Ni.

a [Å]	9.901(5)
b [Å]	9.901(5)
c [Å]	8.355(8)
α [°]	90
β [°]	90
γ [°]	90
Cell volume [Å^3]	819.1(1)
Wavelength [Å]	1.5406
2*θ* range [°]	5-50
R_p	0.0433
R_{wp}	0.0582
R_{exp}	0.0514
Goof	1.131

S3.2. Rietveld and Pawley refinements

Pawley and Rietveld refinements were carried out using the structure analysis software TOPAS (v.6).

S3.2.1. Rietveld refinement

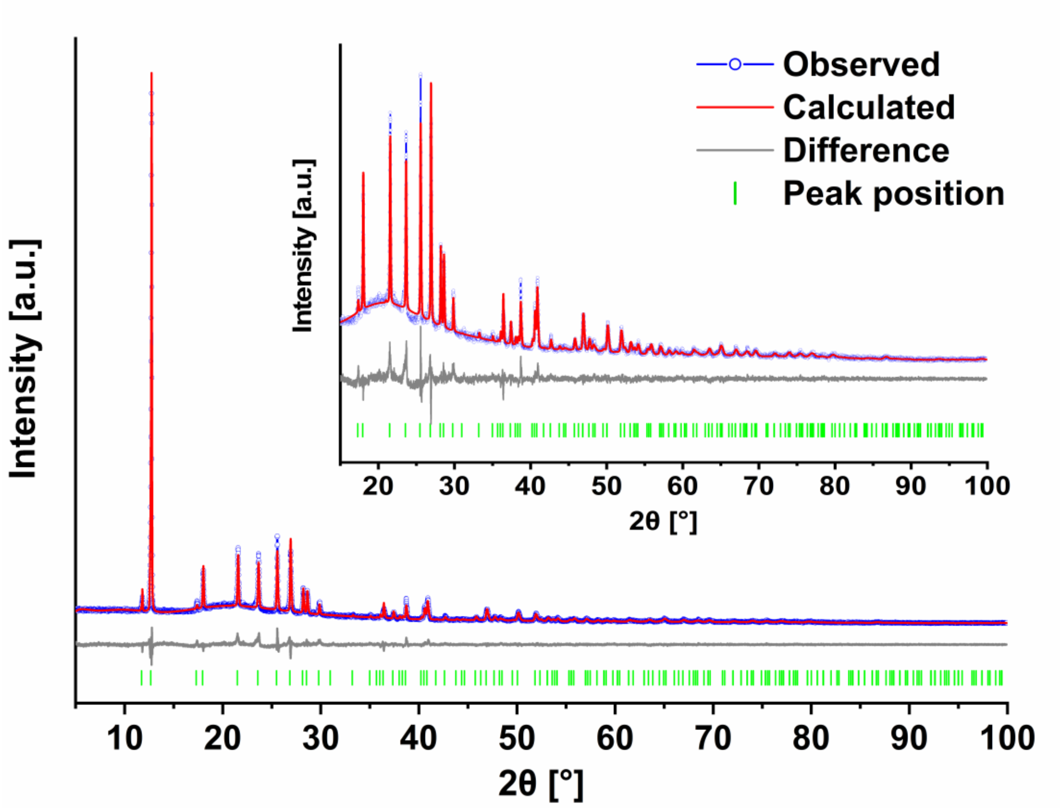

Figure S4. Rietveld refinement plot of VOFFIVE-1-Ni·$2H_2O$ showing the observed (blue), calculated fit (red), difference (gray), and reflections (green) data.

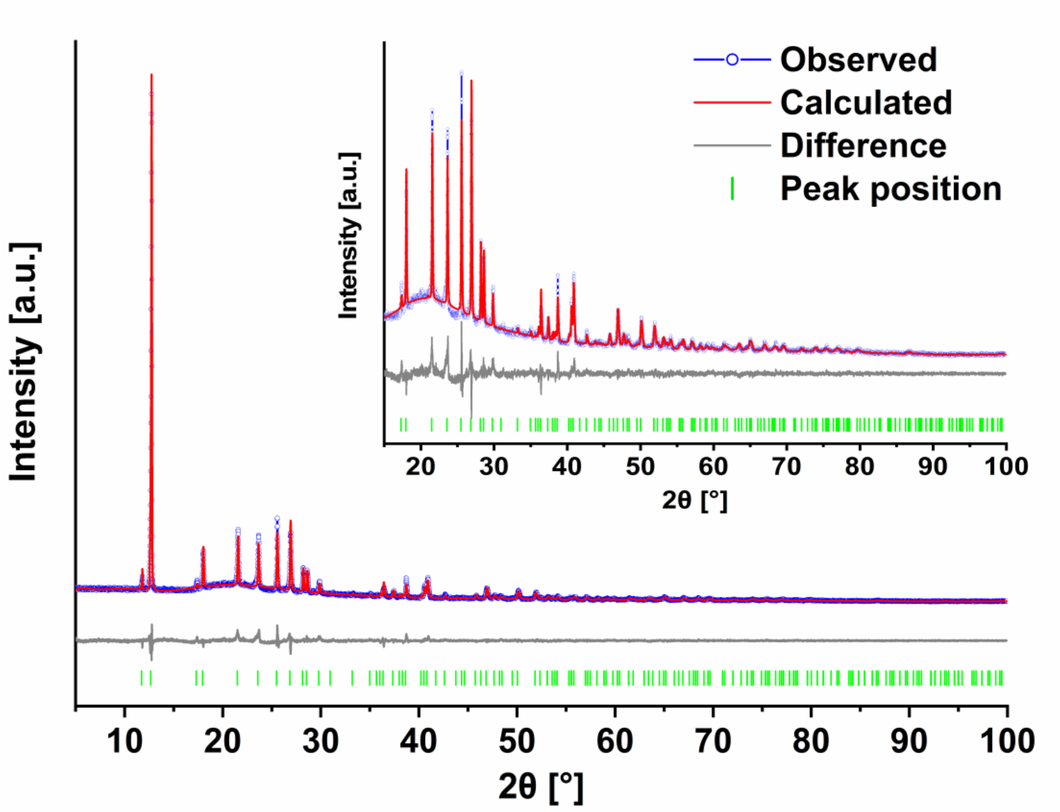

Figure S5. Rietveld refinement plot of NbOFFIVE-1-Ni·CH_3OH showing the observed (blue), calculated fit (red), difference (gray), and reflections (green) data.

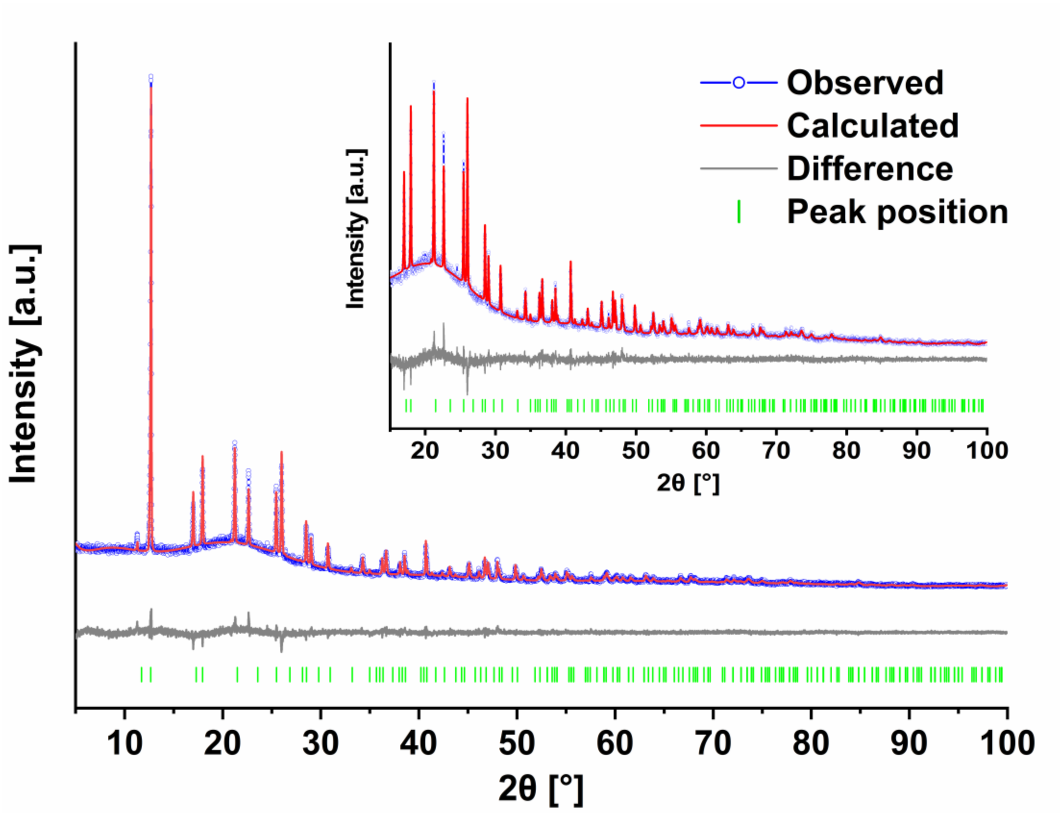

Figure S6. Rietveld refinement plot of TaOFFIVE-1-Ni·CH_3OH showing the observed (blue), calculated fit (red), difference (gray), and reflections (green) data.

Table S3. Crystallographic parameters and details from Rietveld refinement from powder X-ray diffraction data of VOFFIVE-1-Ni·2H_2O, NbOFFIVE-1-Ni·CH_3OH, and TaOFFIVE-1-Ni·CH_3OH.

Compound	VOFFIVE-1-Ni·2H_2O	NbOFFIVE-1-Ni·CH_3OH	TaOFFIVE-1-Ni·CH_3OH
Crystal system	Tetragonal	Tetragonal	Tetragonal
Space group	*I4/mcm* (No. 140)	*I4/mcm* (No. 140)	*I4/mcm* (No. 140)
a [Å]	9.8826(6)	9.9210(6)	9.9249(7)
b [Å]	9.8826(6)	9.9210(6)	9.9249(1)
c [Å]	15.0921(2)	15.7625(3)	15.7633(4)
α [º]	90	90	90
β [º]	90	90	90
γ [º]	90	90	90
V [$Å^3$]	1474.0(1)	1551.4(5)	1552.7(7)
d_{cryst} [cm^3 g^{-1}]	1.8783	1.9429	2.3265
T [K]	293(2)	293(2)	293(2)
Wavelength [Å]	Cu k$\alpha_{1,2}$	Cu k$\alpha_{1,2}$	Cu k$\alpha_{1,2}$
	λ_1 = 1.5406	λ_1 = 1.5406	λ_1 = 1.5406
	λ_2 = 1.5444	λ_2 = 1.5444	λ_2 = 1.5444
R_{wp}	8.592	8.193	8.796
Goof	1.586	1.620	1.686

Table S4. Atomic positions for VOFFIVE-1-Ni·2H_2O obtained from Rietveld refinement.

Atom	Site	x	y	z	Occupancy	U_{iso} (Å^2)
C	C1A	0.2465	0.1608	0.3178	1	0.0079
F1/O	F1	0	0	0.1231	0.5	None
F2	F2	-0.0189	0.1896	0	1	0.1465
N	N1	0.1545	0.1545	0.25	1	None
Ni	Ni1	0	0	0.25	1	0.0119
O of H_2O	O1W	0.4365	−0.0635	0.4233	0.5	0.2115
V	V1	0	0	0	1	0.0439

Table S5. Atomic positions for NbOFFIVE-1-Ni·CH_3OH obtained from Rietveld refinement.

Atom	Site	x	y	z	Occupancy	U_{iso} (Å^2)
C	C1A	0.2420	0.1572	0.3145	1	0.0313
C of CH_3OH	C1M	0.5024	0.0174	0.3808	0.1662	0.1900
F1/O	F1	0	0	0.1257	0.5	0.0313
F2	F2	0.0553	0.1828	0	1	0.0313
N	N1	0.1516	0.1516	0.25	1	0.0313
Nb	Nb1	0	0	0	1	0.0228
Ni	Ni1	0	0	0.25	1	0.0133
O of CH_3OH	O1M	0.4103	−0.0858	0.4063	0.1662	0.1900

Table S6. Atomic positions for TaOFFIVE-1-Ni·CH_3OH obtained from Rietveld refinement.

Atom	Site	x	y	z	Occupancy	U_{iso} (Å^2)
C	C1A	0.2399	0.1532	0.3099	1	None
C of CH_3OH	C1M	0.4930	0.0102	0.3945	0.156	0.1900
F1/O	F1	0	0	0.1287	0.5	0.0130
F2	F2	0.04445	0.1855	0	1	0.0410
N	N1	0.1499	0.1499	0.25	1	None
Ni	Ni1	0	0	0.25	1	None
O of CH_3OH	Ni1	0.4030	−0.1020	0.3879	0.156	0.1900
Ta	Ta1	0	0	0	1	0.0246

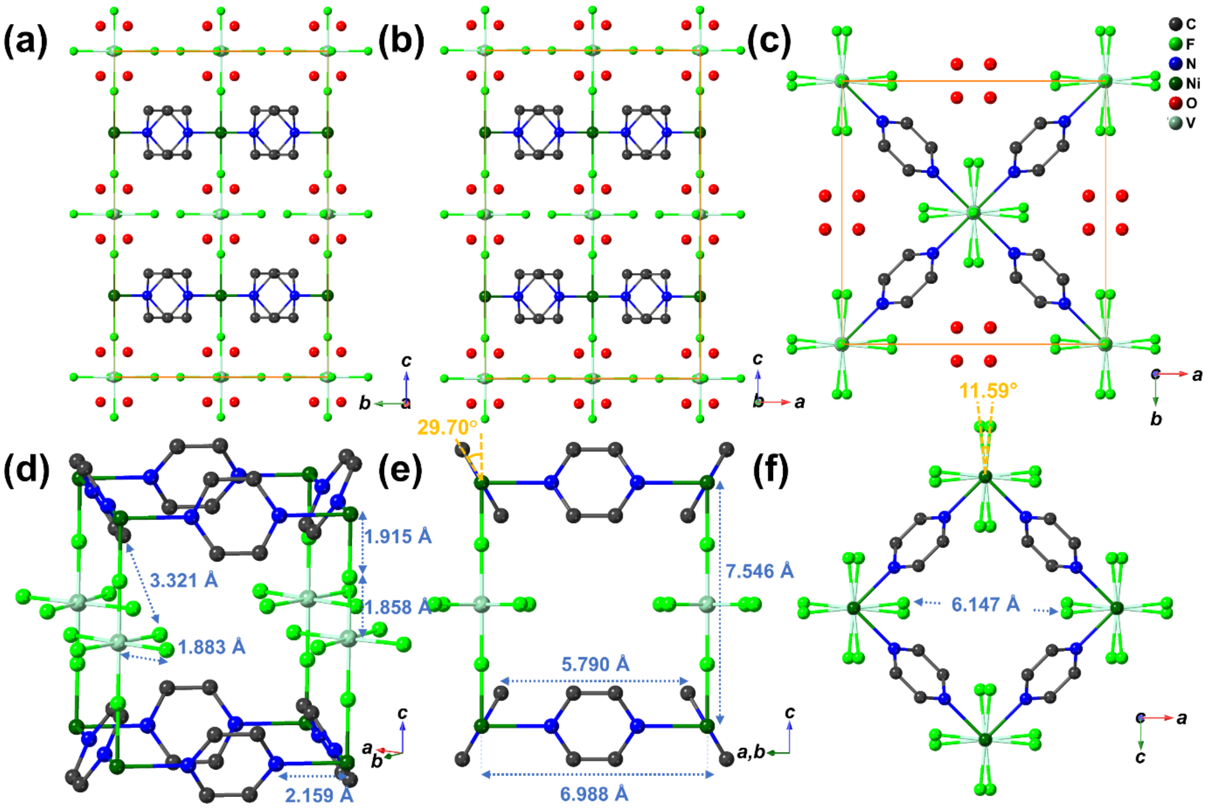

Figure S7. Crystal structure of VOFFIVE-1-Ni·2H_2O as shown along (a) [100], (b) [010], (c) [001], (d) bond lengths and atomic distances, (e) tilting of the pyz-rings from the c-axis, and (f) rotational displacement of F-moieties in the anionic inorganic unit between two adjacent layers. The outline of the unit cell in (a) – (c) is highlighted in orange and solvent molecules were omitted in (d) – (f) for clarity.

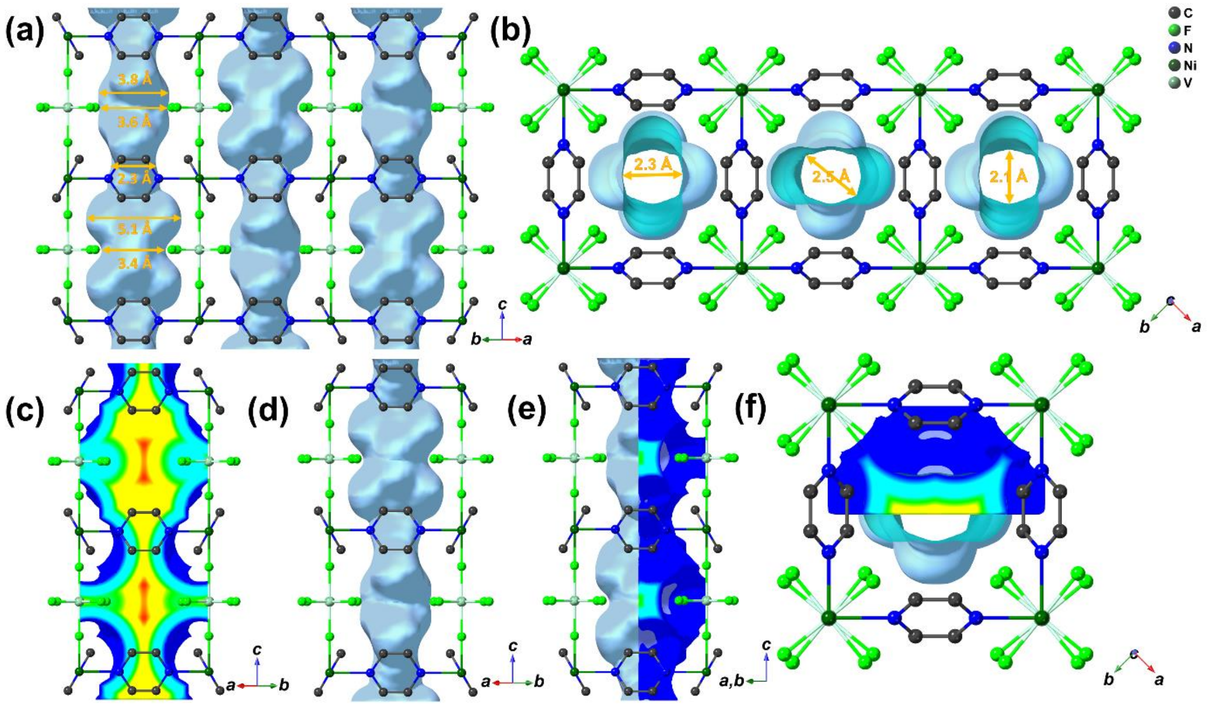

Figure S8. Crystal structure of as-synthesized VOFFIVE-1-Ni·$2H_2O$ (solvent molecules excluded) showing the (a) solvent-excluded pore surface obtained using a probe radius of 1.2 Å, (b) the pore surface as viewed along [001] (the light blue and turquoise colors represent the exterior and interior pore surface, respectively), (c) a contour map displaying the distance between the atoms in the pore channels (gradient from dark blue to red denote an increasing interatomic distance), (d) corresponding pore surface viewed in the same direction as (c), (e) pore surface and interatomic distances viewed perpendicular to the viewing direction in (c – d), and (f) pore surface and interatomic distances viewed along [001].

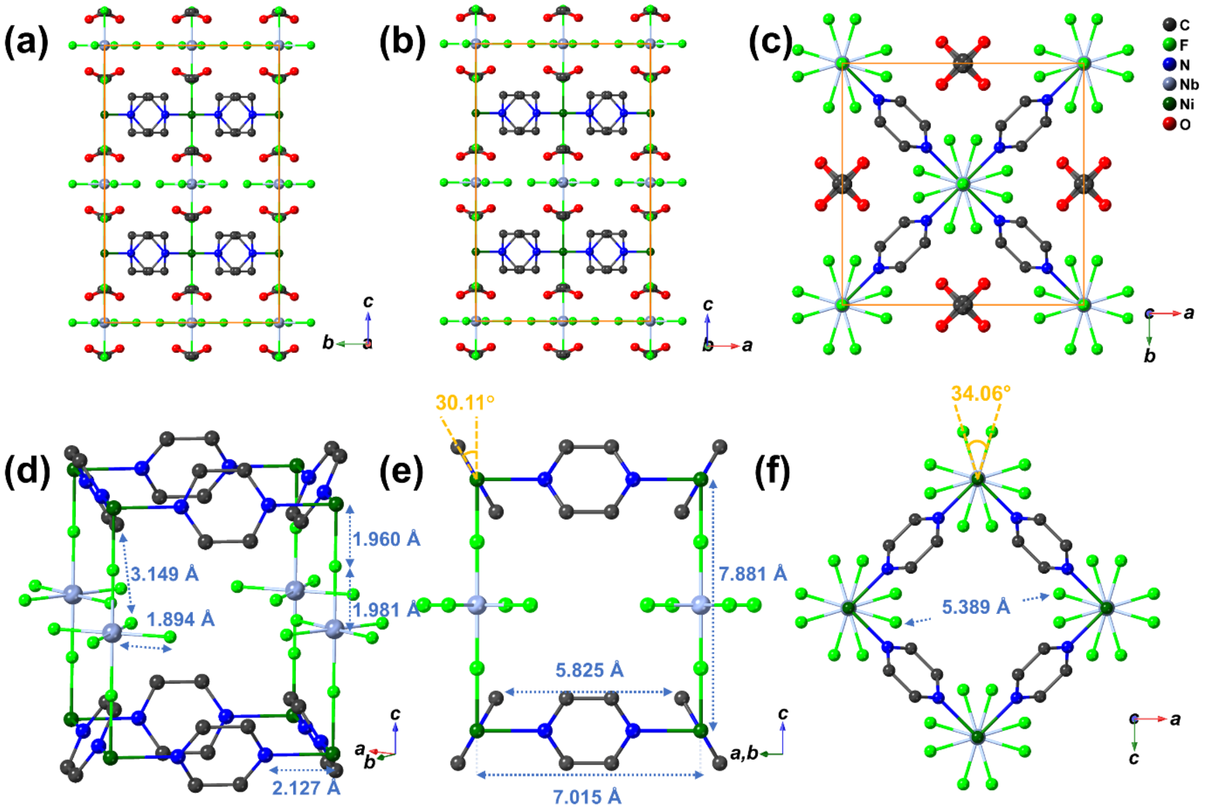

Figure S9. Crystal structure of NbOFFIVE-1-Ni·CH_3OH as shown along (a) [100], (b) [010], (c) [001], (d) bond lengths and atomic distances, (e) tilting of the pyz-rings from the c-axis, and (f) rotational displacement of F-moieties in the anionic inorganic unit between two adjacent layers. The outline of the unit cell in (a) – (c) is highlighted in orange and solvent molecules were omitted in (d) – (f) for clarity.

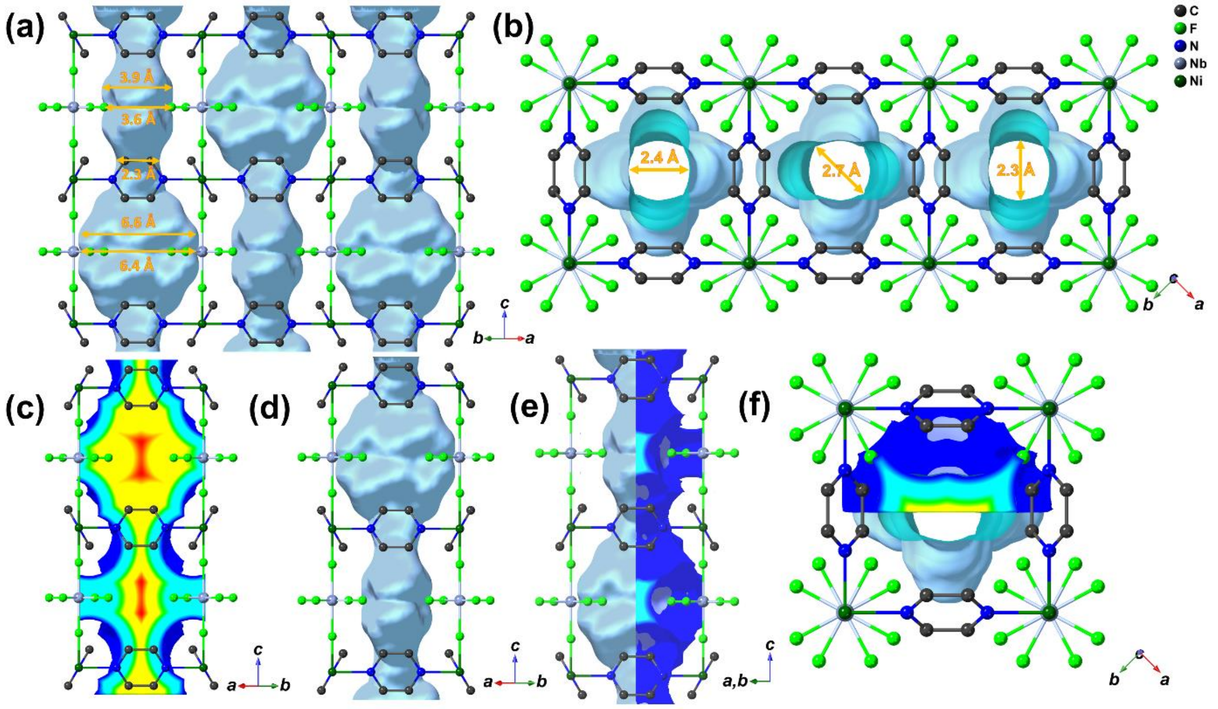

Figure S10. Crystal structure of as-synthesized NbOFFIVE-1-Ni·CH_3OH (solvent molecules excluded) showing the (a) solvent-excluded pore surface obtained using a probe radius of 1.2 Å, (b) the pore surface as viewed along [001] (the light blue and turquoise colors represent the exterior and interior pore surface, respectively), (c) a contour map displaying the distance between the atoms in the pore channels (gradient from dark blue to red denote an increasing interatomic distance), (d) corresponding pore surface viewed in the same direction as (c), (e) pore surface and interatomic distances viewed perpendicular to the viewing direction in (c – d), and (f) pore surface and interatomic distances viewed along [001].

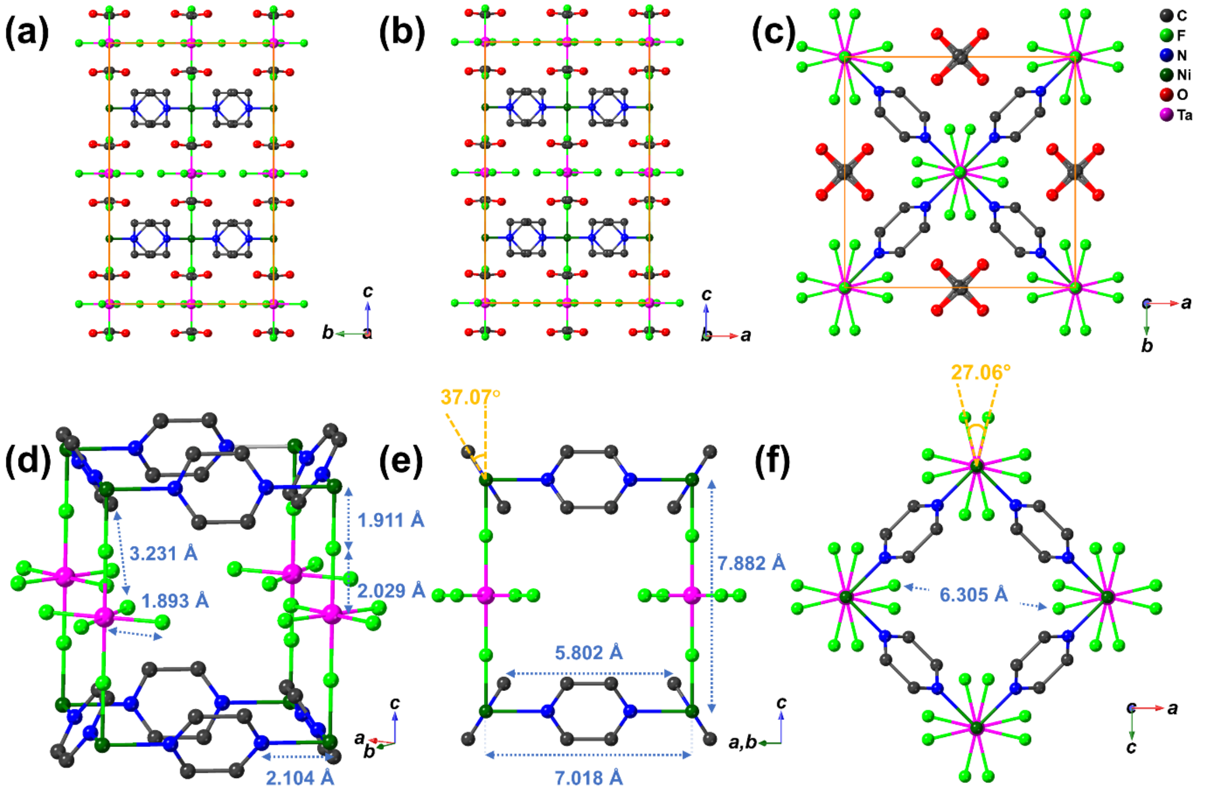

Figure S11. Crystal structure of TaOFFIVE-1-Ni·CH_3OH as shown along (a) [100], (b) [010], (c) [001], (d) bond lengths and atomic distances, (e) tilting of the pyz-rings from the c-axis, and (f) rotational displacement of F-moieties in the anionic inorganic unit between two adjacent layers. The outline of the unit cell in (a) – (c) is highlighted in orange and solvent molecules were omitted in (d) – (f) for clarity.

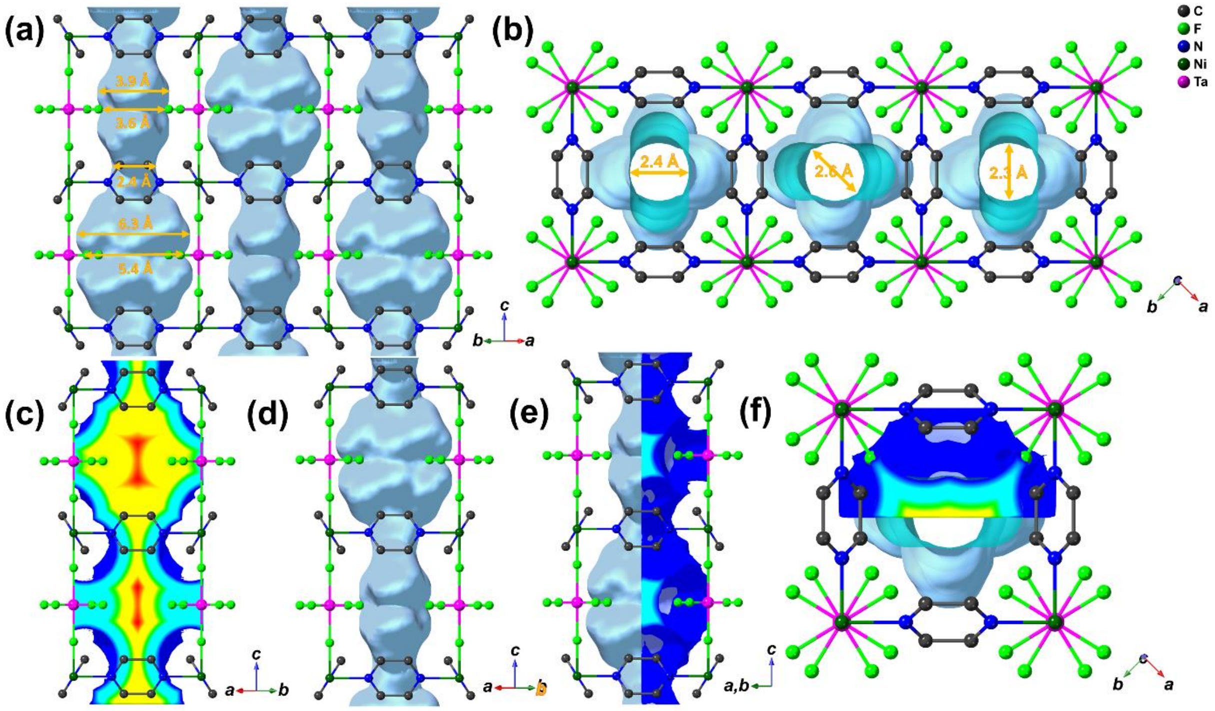

Figure S12. Crystal structure of as-synthesized TaOFFIVE-1-Ni·CH_3OH (solvent molecules excluded) showing the (a) solvent-excluded pore surface obtained using a probe radius of 1.2 Å, (b) the pore surface as viewed along [001] (the light blue and turquoise colors represent the exterior and interior pore surface, respectively), (c) a contour map displaying the distance between the atoms in the pore channels (gradient from dark blue to red denote an increasing interatomic distance), (d) corresponding pore surface viewed in the same direction as (c), (e) pore surface and interatomic distances viewed perpendicular to the viewing direction in (c – d), and (f) pore surface and interatomic distances viewed along [001].

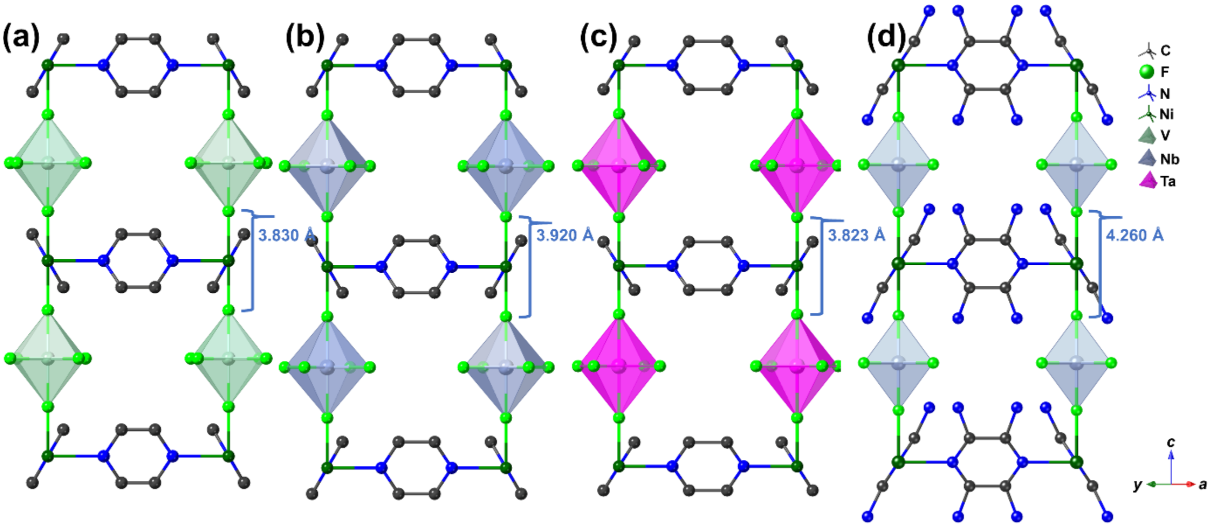

Figure S13. Projection of the crystals structure of (a) VOFFIVE-1-Ni·$2H_2O$, (b) NbOFFIVE-1-Ni·CH_3OH, (c) TaFFIVE-1-Ni·CH_3OH, and (d) NbOFFIVE-2-Ni showing the distance between adjacent anionic pillars. Solvent molecules are omitted for clarity.

S3.2.2. Pawley refinement

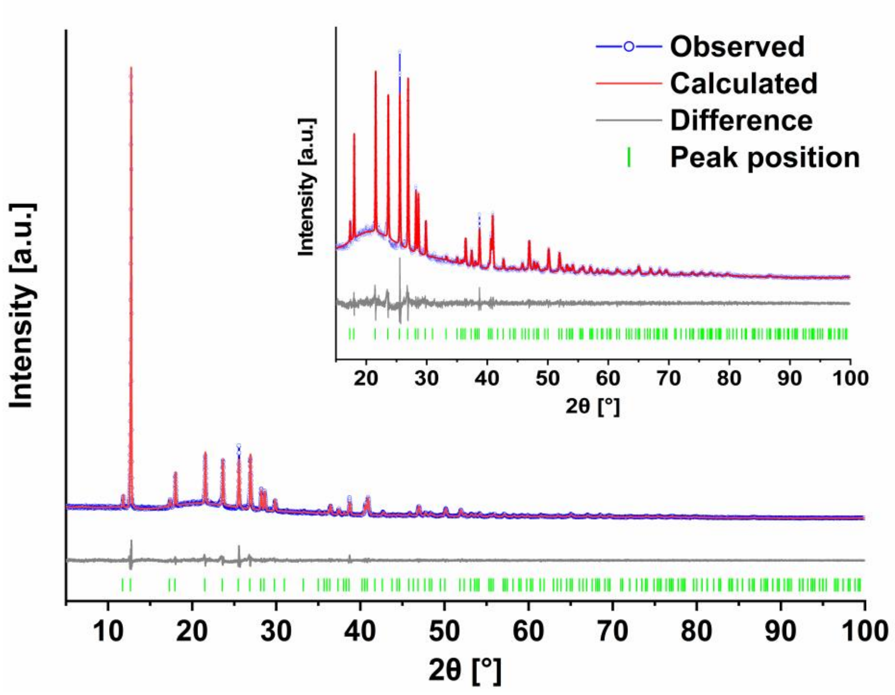

Figure S14. Observed (blue), Pawley fit (red), difference (gray), and reflections (green) of VOFFIVE-1-Ni·$2H_2O$.

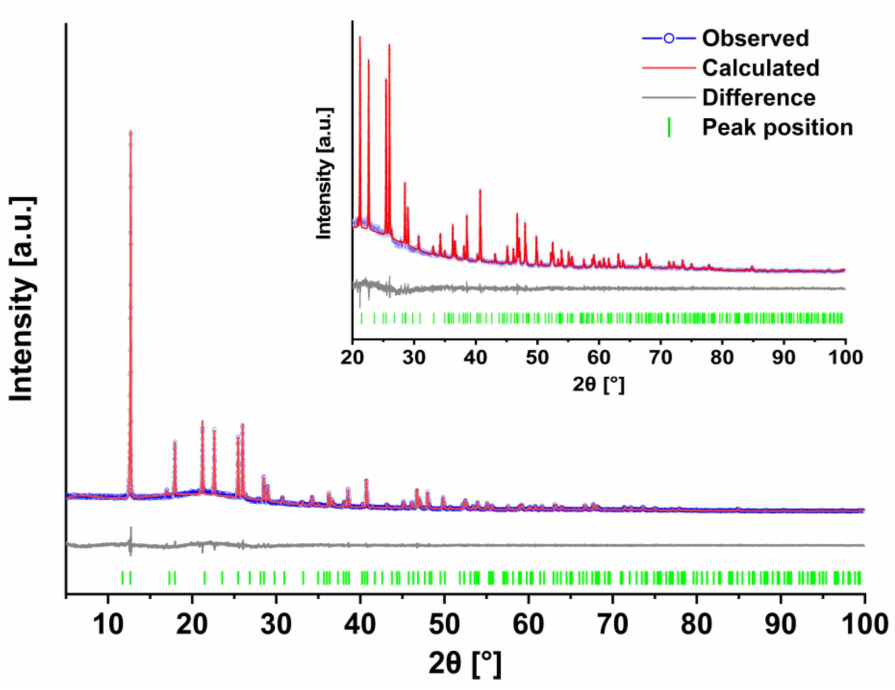

Figure S15. Observed (blue), Pawley fit (red), difference (gray), and reflections (green) of NbOFFIVE-1-Ni·CH_3OH.

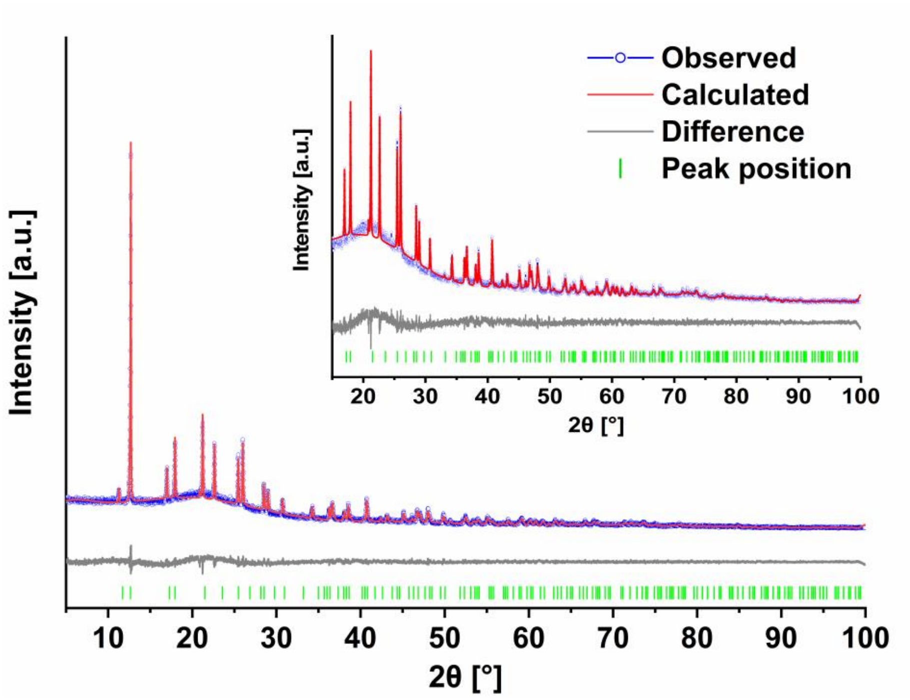

Figure S16. Observed (blue), Pawley fit (red), difference (gray), and reflections (green) of TaOFFIVE-1-Ni·CH_3OH.

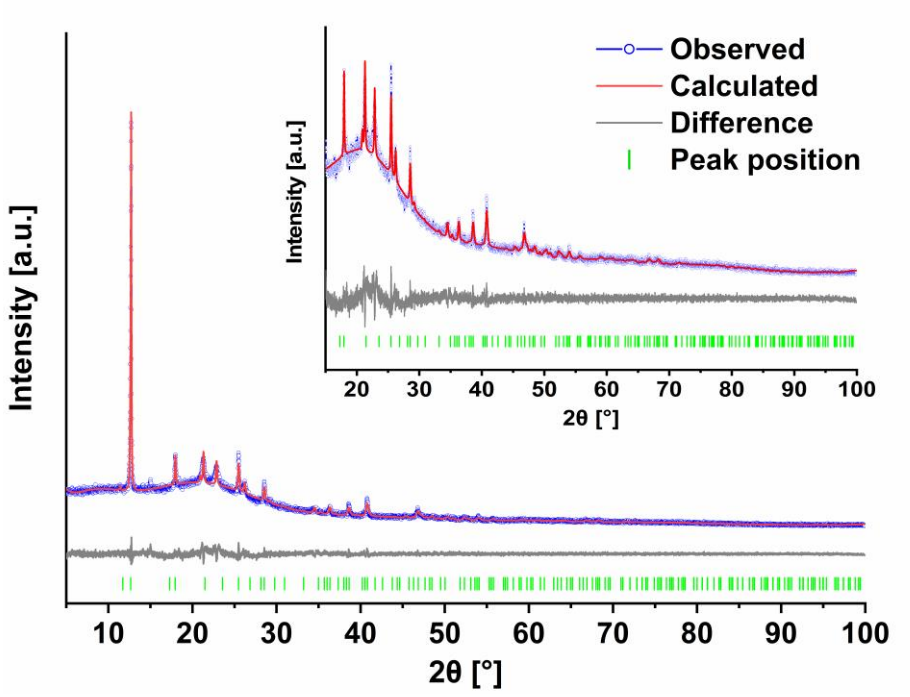

Figure S17. Observed (blue), Pawley fit (red), difference (gray), and reflections (green) of NbOFFIVE-2-Ni.

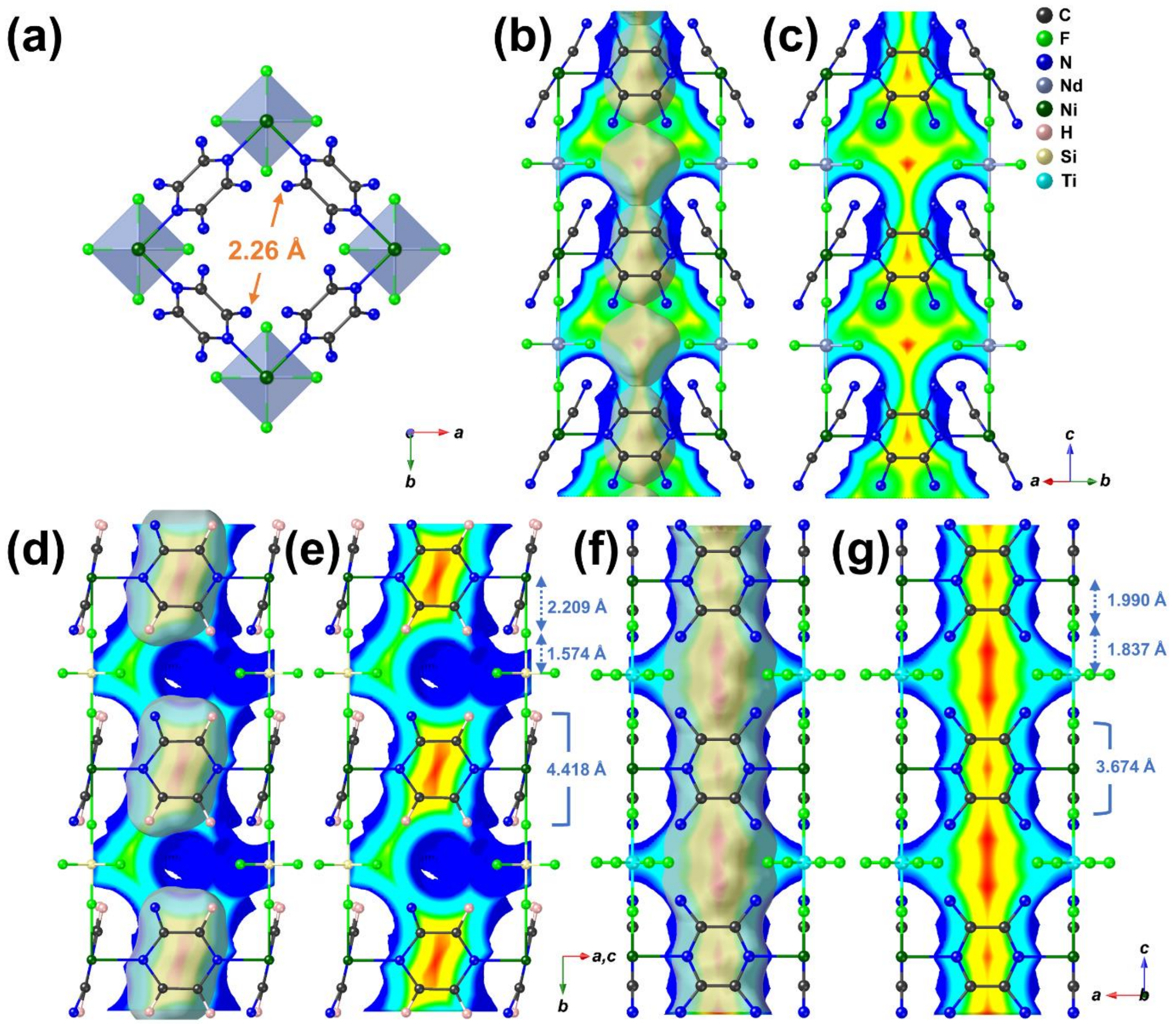

Figure S18. (a) crystal structure of NbOFFIVE-2-Ni as viewed along [001] showing the placement of the pyz-NH_2 ligands and anionic $(NbOF_5)^{2-}$ pillars as well as pyz-NH_2-limiting pore aperture, (b) solvent-excluded surface (obtained using a probe radius of 1.2 Å) superimposed onto the cross section of a distance map (excluding the van der Waals radii of the atoms) showing the interior of the pore cavity (the -NH_2 group is disordered over four positions on the pyz-NH_2 ligand).

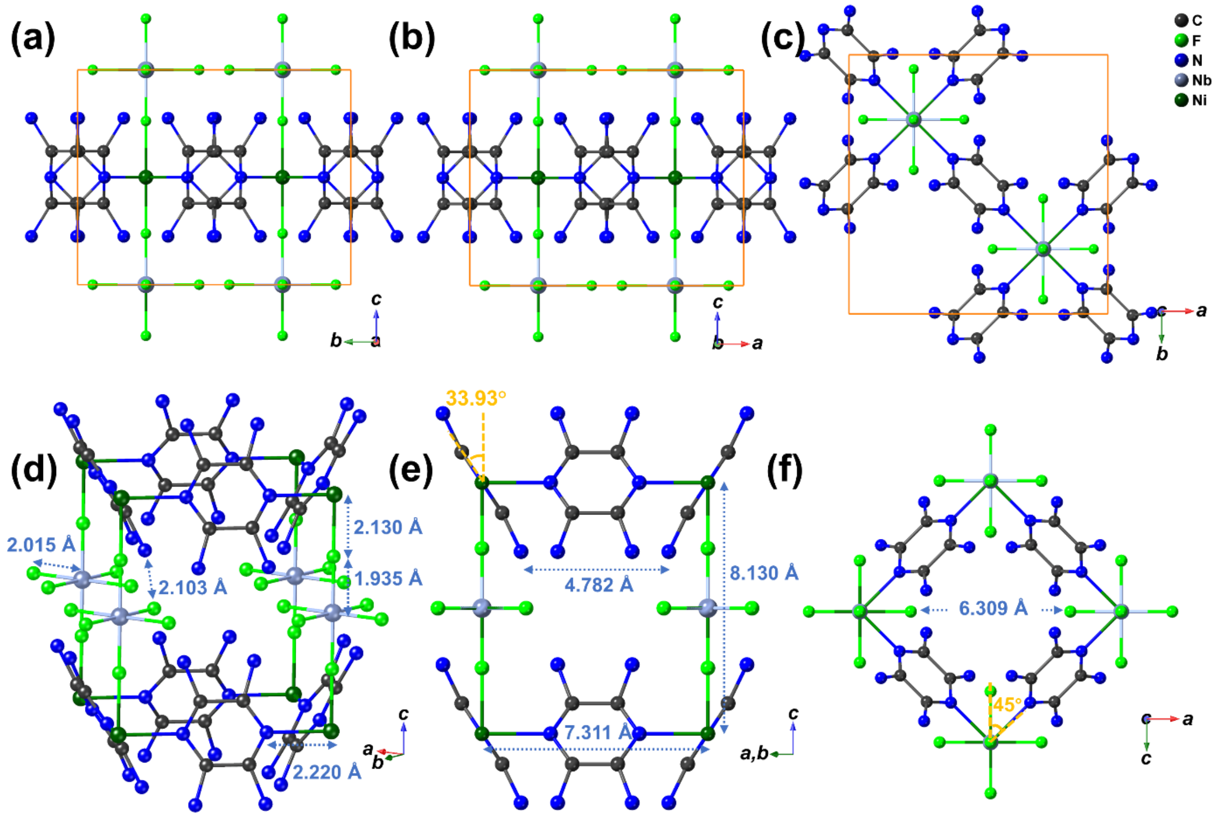

Figure S19. Crystal structure of NbOFFIVE-2-Ni obtained from Pawley refined 3D ED data as shown along (a) [100], (b) [010], (c) [001], (d) bond lengths and atomic distances, (e) tilting of the pyz-NH_2-rings from the c-axis, and (f) rotational displacement of F-moieties in the anionic inorganic unit between two adjacent layers. The outline of the unit cell in (a) – (c) is highlighted in orange.

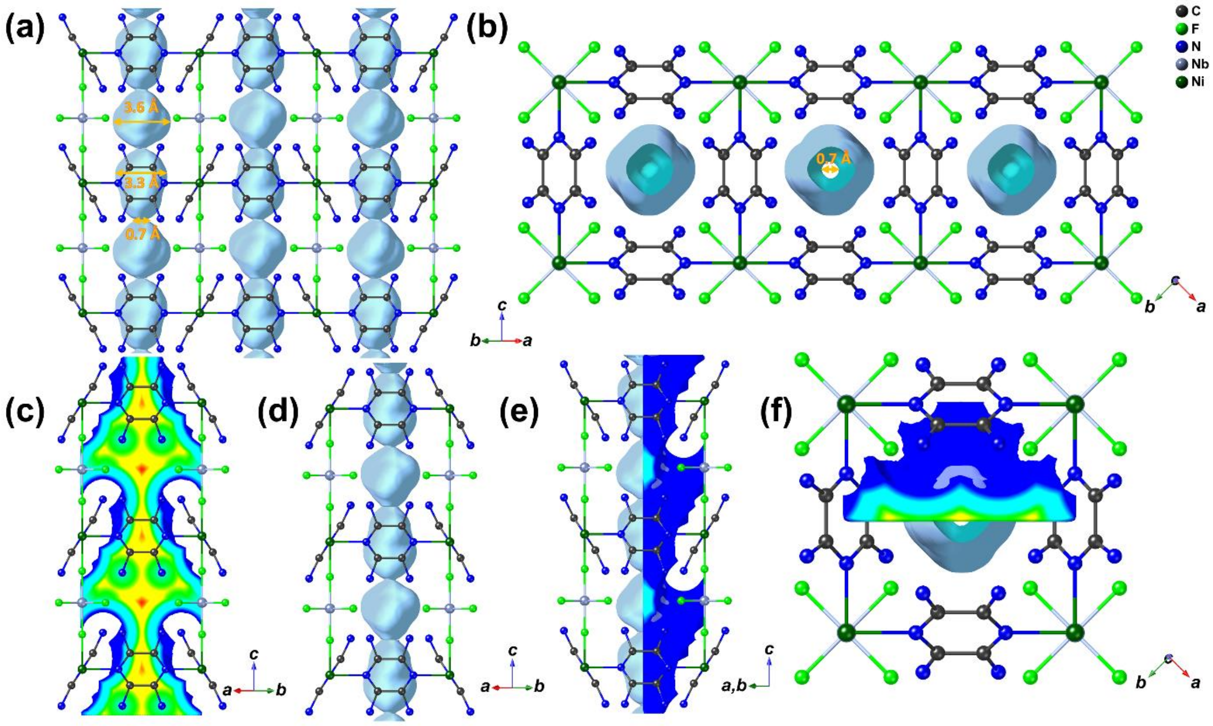

Figure S20. Crystal structure of as-synthesized NbOFFIVE-2-Ni showing the (a) solvent-excluded pore surface obtained using a probe radius of 1.2 Å, (b) the pore surface as viewed along [001] (the light blue and turquoise colors represent the exterior and interior pore surface, respectively), (c) a contour map displaying the distance between the atoms in the pore channels (gradient from dark blue to red denote an increasing interatomic distance), (d) corresponding pore surface viewed in the same direction as (c), (e) pore surface and interatomic distances viewed perpendicular to the viewing direction in (c – d), and (f) pore surface and interatomic distances viewed along [001]. The NH_2-group on the 2-aminopyrazine ligand is disordered between four positions.

Table S6. Unit cell parameters and interatomic distances of as-synthesized HUM compounds.

	VOFFIVE-1-Ni·2H_2O	NbFFIVE-1-Ni·CH_3OH	TaOFFIVE-1-Ni·CH_3OH
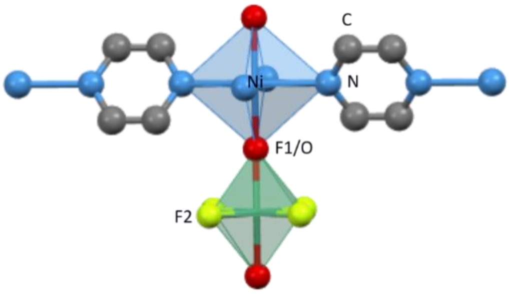			
a=*b* [Å]	9.883	9.921	9.925
c [Å]	15.092	15.763	15.763
$d_{M\text{-}F1}$ [Å]	1.8578	1.9809	1.9427
$d_{M\text{-}F2}$ [Å]	1.8825	1.8944	2.0296
$d_{F1\text{-}Ni}$ [Å]	1.9152	1.9598	1.9115
$d_{Ni\text{-}N}$ [Å]	2.1589	2.1272	2.1042

S3.3. Stability study

S3.3.1. Thermal decomposition

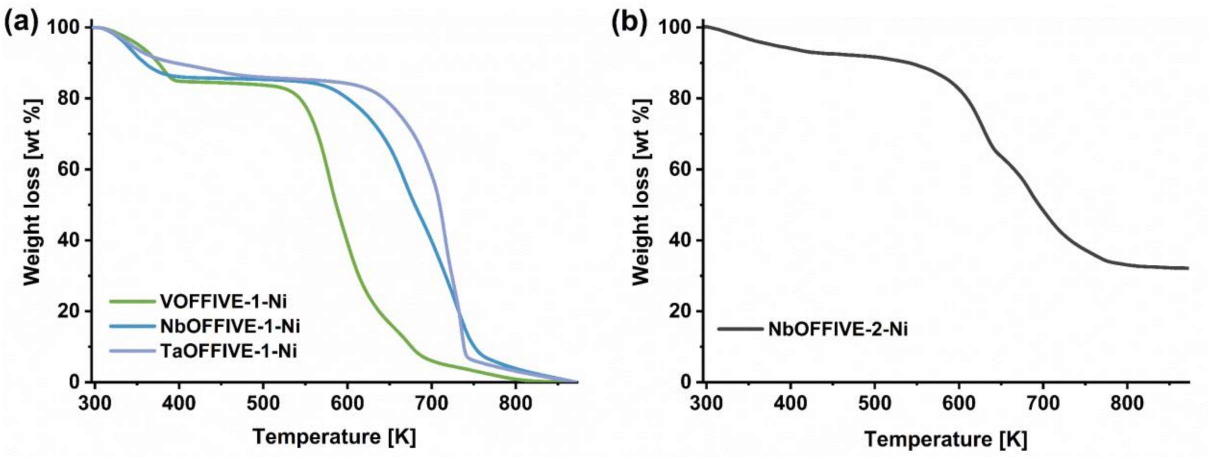

Figure S21. Thermogravimetric decomposition profiles of as-synthesized (a) pyz-based and (b) pyz-NH_2-based HUMs in air.

S3.3.2. Hydrolytic stability

The hydrolytic stability of the as-synthesized HUMs was investigated by stirring the materials (approx. 0.01 g) in 15 ml of deionized water for 3 months, after which the crystallinity of the samples was evaluated by powder X-ray diffraction.

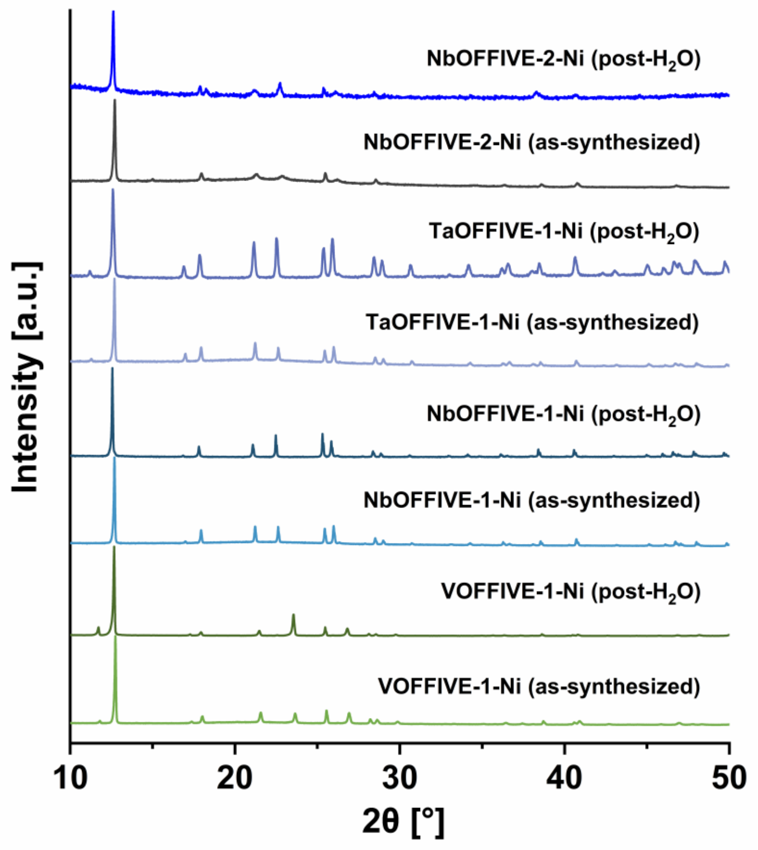

Figure S22. Powder X-ray diffractograms of as-synthesized HUM samples after hydrolytic stability test.

S3.4. Gravimetric CO_2 adsorption cycles and sorbent regeneration

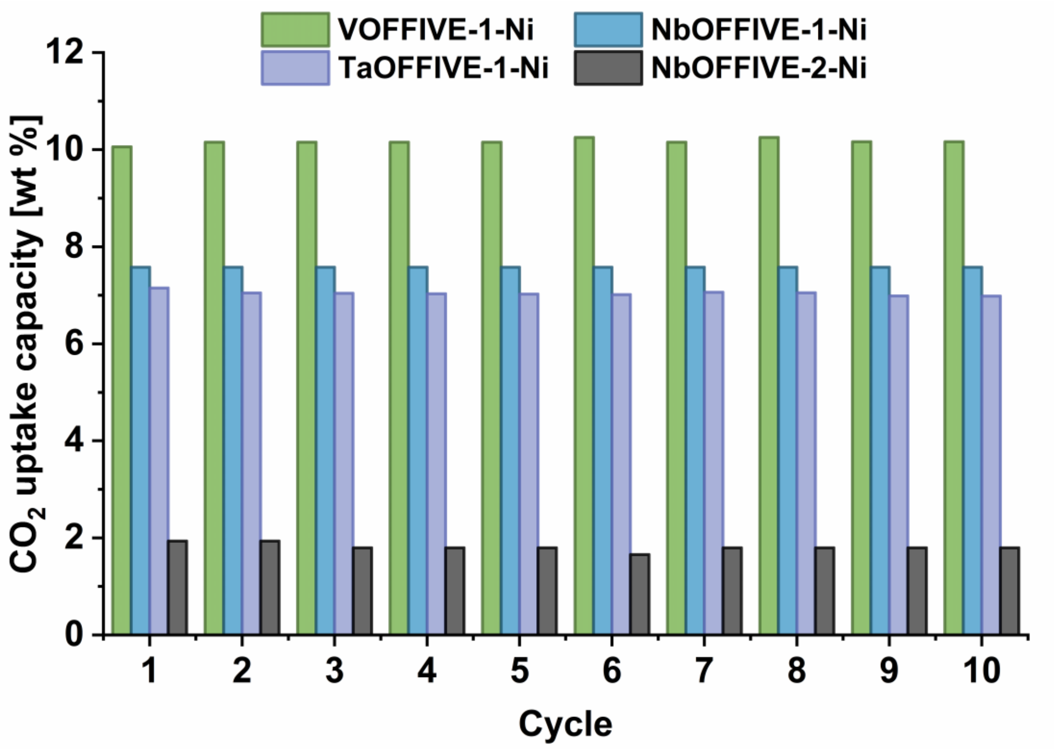

Figure S23. Cyclic gravimetric CO_2 uptake stability at 303 K (50 ml min^{-1} CO_2 flow rate) of isoreticular VOFFIVE-1-Ni, NbOFFIVE-1-Ni, TaOFFIVE-1-Ni, and NbOFFIVE-2-Ni.

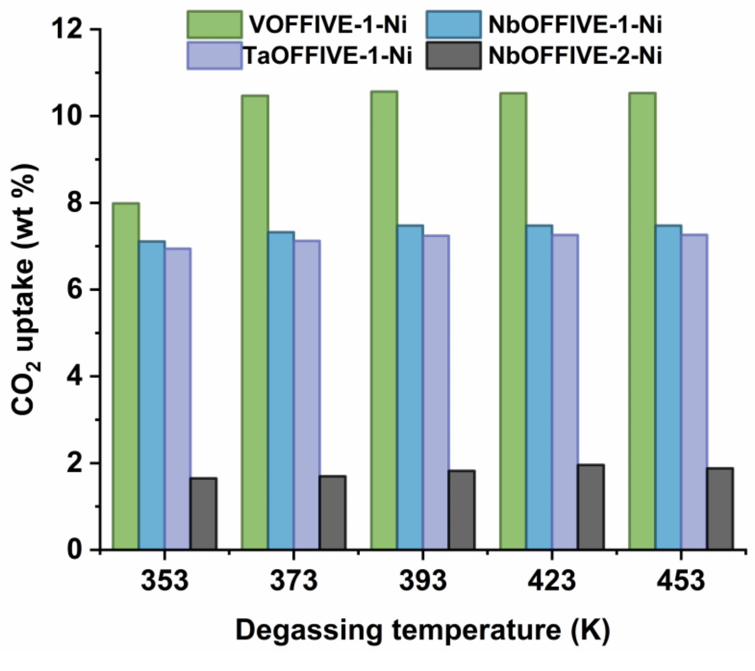

Figure S24. Gravimetric CO_2 uptake at 303 K (50 ml min^{-1} CO_2 flow rate) of VOFFIVE-1-Ni, NbOFFIVE-1-Ni, TaOFFIVE-1-Ni, and NbOFFIVE-2-Ni after degassing at 353 – 435 K for 10 min (50 ml min^{-1} N_2 flow rate).

S3.5. Surface area and porosity

Manometric gas sorption isotherms were recorded on a Micromeritics ASAP 2020 Surface Area and Porosity Analyzer (Norcross, GA, USA) on samples activated at 393.15 K for 3 h under dynamic vacuum (1×10^{-4} mmHg). The Brunauer-Emmett-Teller (BET) surface areas of the HUMs were calculated with the aid of the BET Surface Identification (BETSI) package[10] using the adsorption branch of the N_2 sorption isotherms recorded at 77 K in the relative pressure ranges specified in Figures S25 – S28. Sorption isotherms up to 1 bar or p/p° ≈ 1 of N_2, CO_2, and H_2O at 293 K or at 293 K and 303 K (used to calculate the isosteric enthalpies of CO_2 adsorption) were furthermore collected using a temperature-controlled water bath. The isosteric enthalpy of CO_2 adsorption (ΔH_{ads}) was furthermore calculated from the adsorption branch of the CO_2 sorption isotherms using the Clausius-Clapeyron equation (Equation 1).

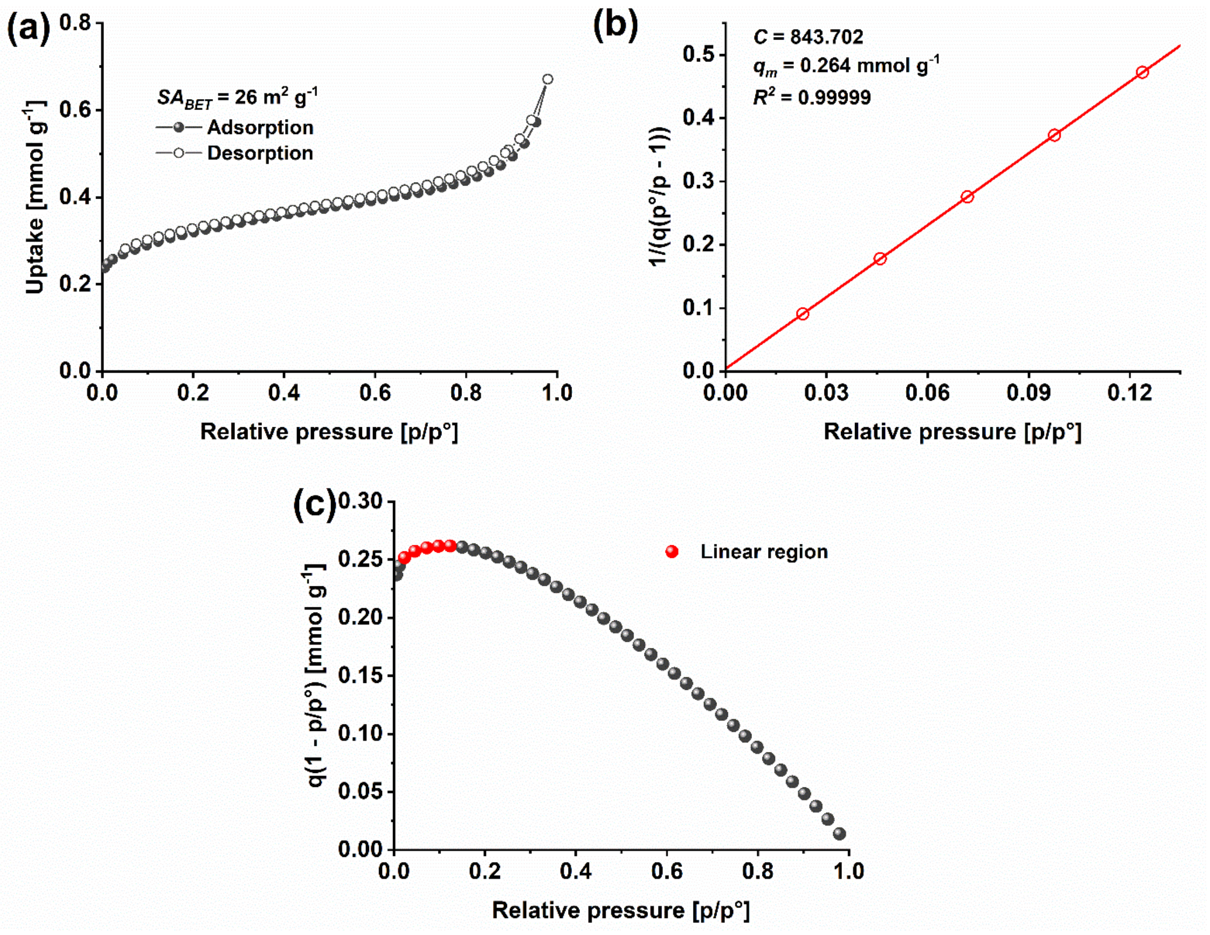

Figure S25. (a) N_2 sorption isotherm of VOFFIVE-1-Ni recorded at 77 K (filled and open circles represent the adsorption and desorption branches, respectively), showing a typical type II non-porous material (b) BET plot showing the linear region wherein the BET model would be valid for porous materials, and (c) Rouquerol plot (the linear region is highlighted in red). BET model was applied to the data here for consistency and quantitative comparison with other samples.

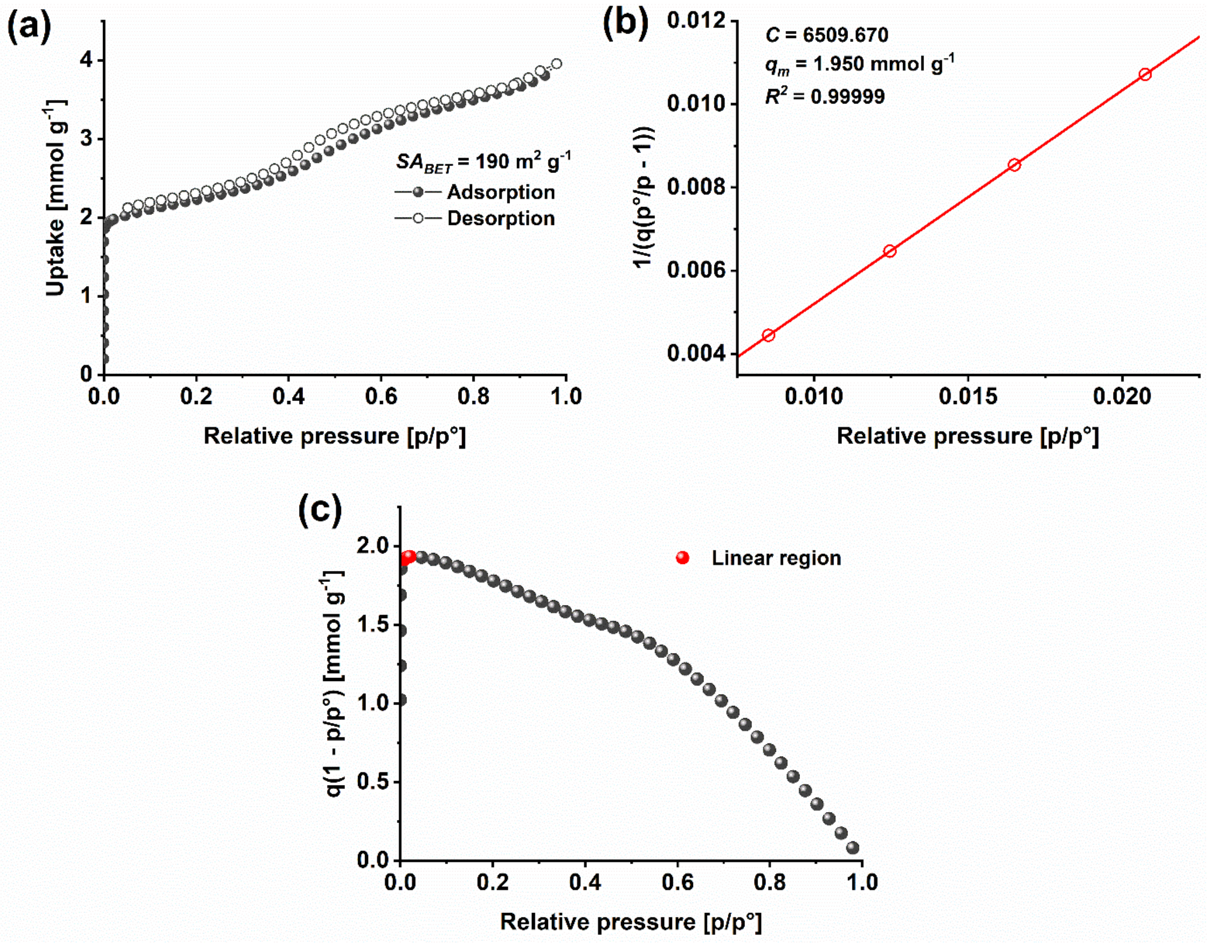

Figure S26. (a) N_2 sorption isotherm of NbOFFIVE-1-Ni recorded at 77 K (filled and open circles represent the adsorption and desorption branches, respectively), showing properties of a type I isotherm for microporous materials, stepwise isotherm shape at p/p^0~ 0.5 may be related to changes in the structure at high N_2 coverage, (b) BET plot showing the linear region wherein the BET model is valid, and (c) Rouquerol plot (linear region is highlighted in red).

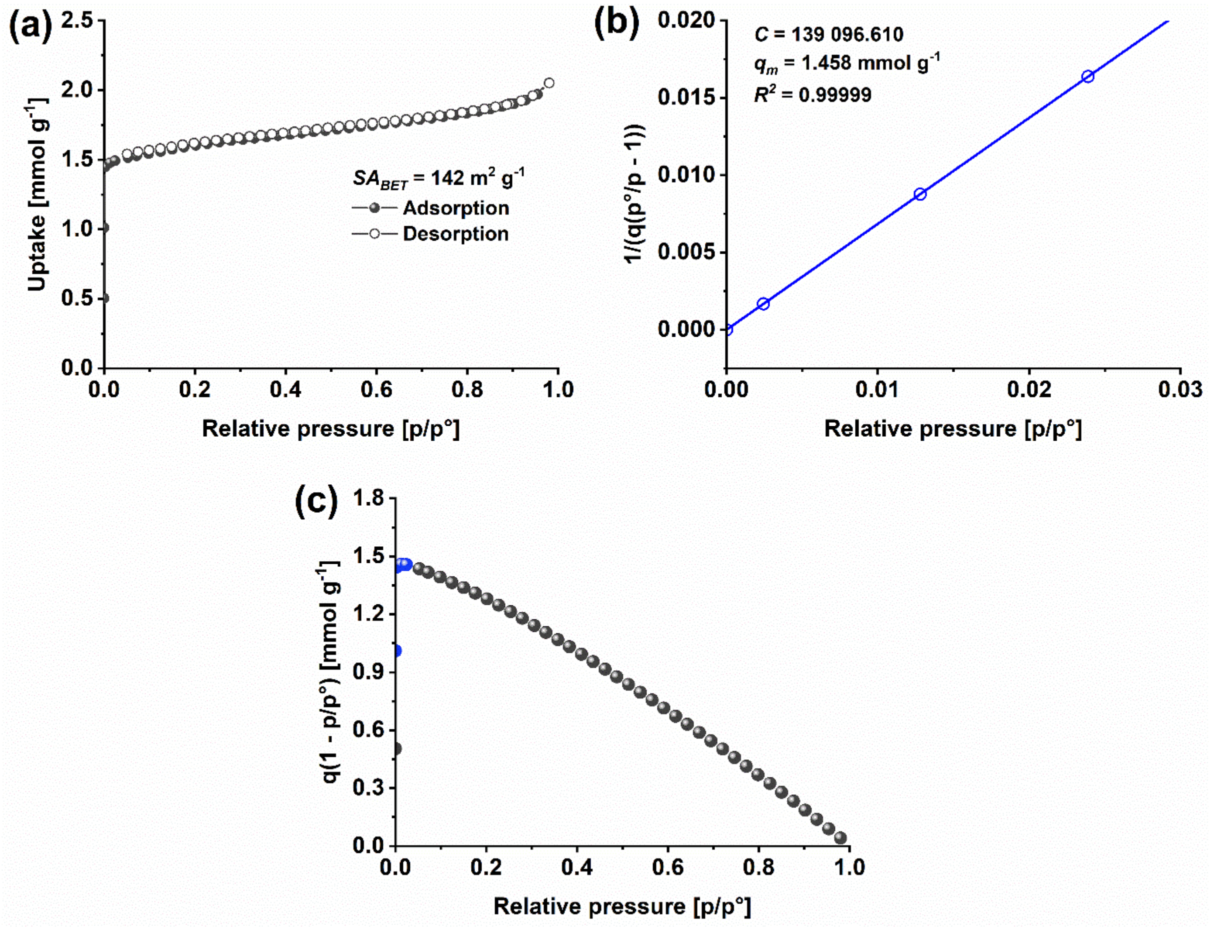

Figure S27. (a) N_2 sorption isotherm of TaOFFIVE-1-Ni recorded at 77 K (filled and open circles represent the adsorption and desorption branches, respectively), showing a typical type I isotherm for microporous materials (b) BET plot showing the chosen region used for calculating the BET surface area, and (c) Rouquerol plot (chosen region highlighted in blue).

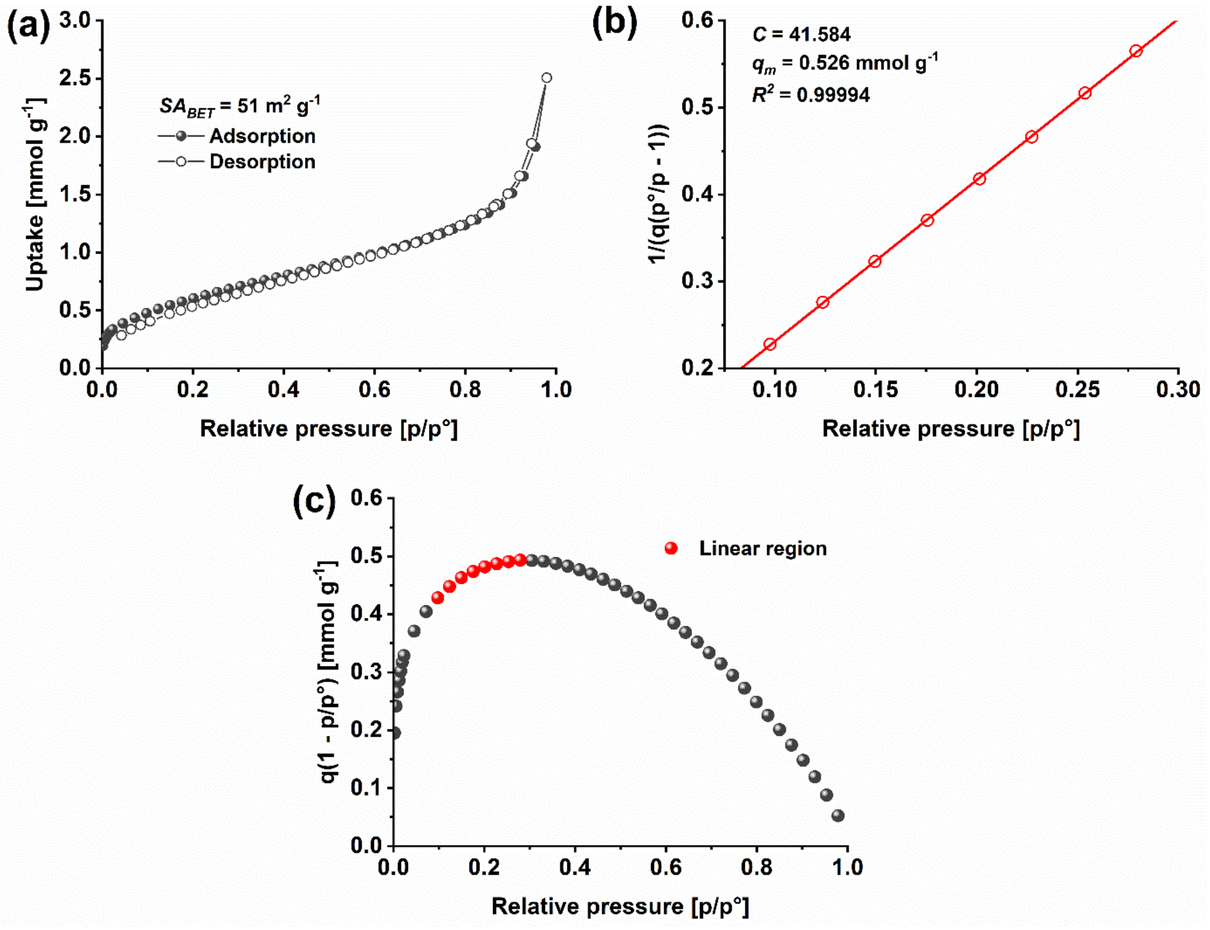

Figure S28. (a) N_2 sorption isotherm of NbOFFIVE-2-Ni recorded at 77 K (filled and open circles represent the adsorption and desorption branches, respectively), showing a typical type II non-porous material (b) BET plot showing the linear region wherein the BET model would be valid for porous materials, and (c) Rouquerol plot (the linear region is highlighted in red). BET model was applied to the data here for consistency and quantitative comparison with other samples.

S3.5.1. CO_2, N_2, and CH_4 sorption isotherms

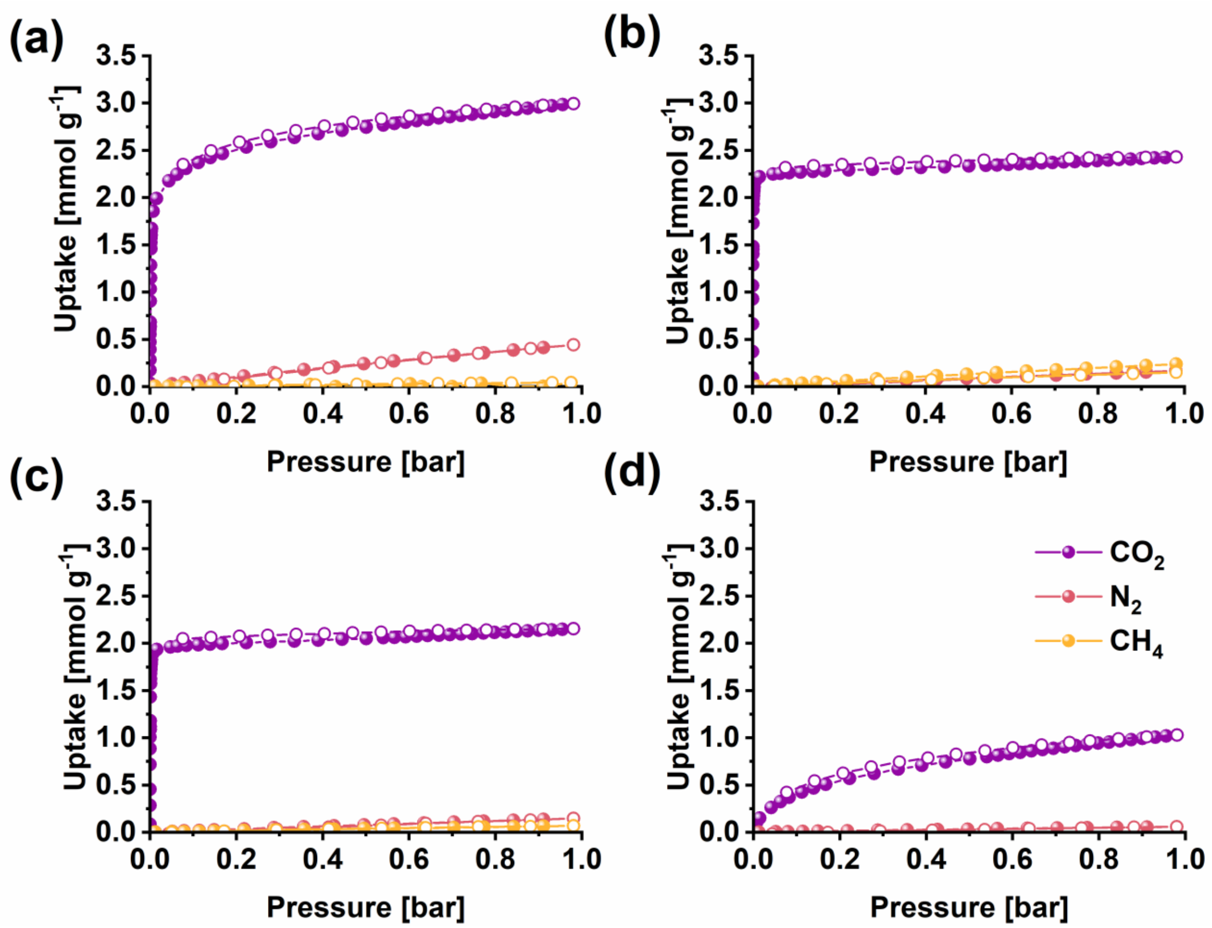

Figure S29. CO_2, N_2, and CH_4 sorption isotherm recorded at 293 K for (a) VOFFIVE-1-Ni, (b) NbOFFIVE-1-Ni, (c) TaOFFIVE-1-Ni, and (d) NbOFFIVE-2-Ni.

S3.5.2. Isosteric enthalpies of CO_2 adsorption

Isosteric enthalpies of CO_2 adsorption (ΔH_{ads}) were calculated from CO_2 adsorption isotherms recorded at 293 K and 303 K within a pressure range of 0.19 – 1.49 mbar using the Clausius-Clapeyron equation[11]:

$$\Delta H_{ads} = -R \left(\frac{\partial\, p}{\partial\, T^{-1}} \right)_q \qquad \text{Equation 1}$$

Where R [J mol^{-1} K^{-1}] is the ideal gas constant, p [bar] pressure, T [K] temperature, and q [mmol g^{-1}] coverage.

All CO_2 isotherms were fitted using the Langmuir-Freundlich model:

$$q_{eq} = q_{sat} \frac{(bp)^a}{1+(bp)^a} \qquad \text{Equation 2}$$

Where q_{eq} [mmol g^{-1}] is the equilibrium CO_2 uptake at pressure p [bar], q_{sat} [mmol g^{-1}] the saturation uptake capacity of an adsorption site, b the affinity parameter for the specific adsorption site.

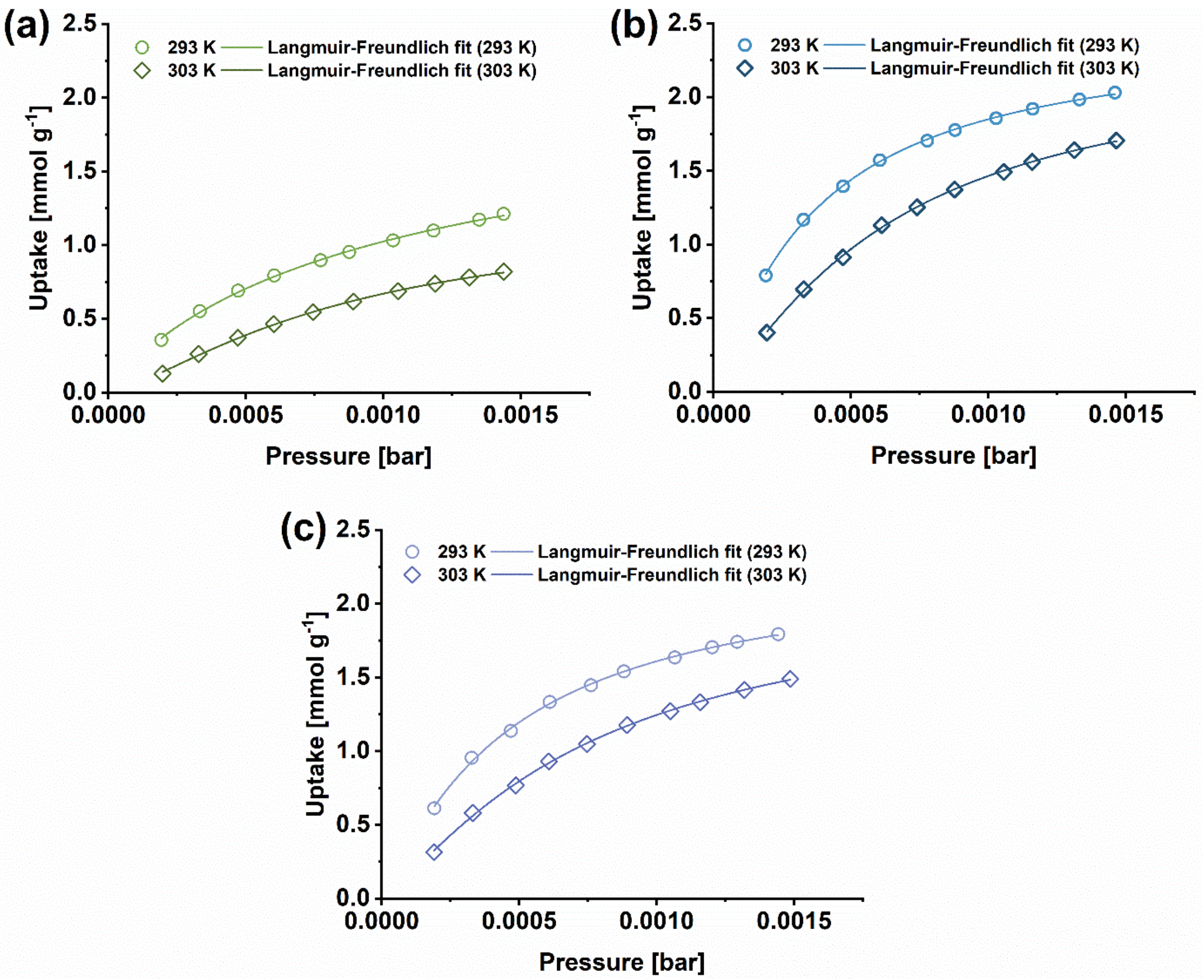

Figure S30. CO_2 adsorption isotherms recorded at 293 and 303 K and 0.19 – 1.49 mbar fitted using the Langmuir-Freundlich model for (a) VOFFIVE-1-Ni, (b) NbOFFIVE-1-Ni, and (c) TaOFFIVE-1-Ni.

Table S7. Fitted Langmuir-Freundlich parameters.

Compound	**VOFFIVE-1-Ni**		**NbOFFIVE-1-Ni**		**TaOFFIVE-1-Ni**	
T [K]	293	303	293	303	293	303
q_{sat} [mmol g^{-1}]	2.179	1.209	2.450	2.272	2.296	2.130
b [bar^{-1}]	874.54	1170.74	2743.28	1588.40	2155.11	1317.72
a	0.892	1.390	1.115	1.288	1.110	1.238
RMSE	0.01037	0.00684	0.01075	0.01032	0.01449	0.01088

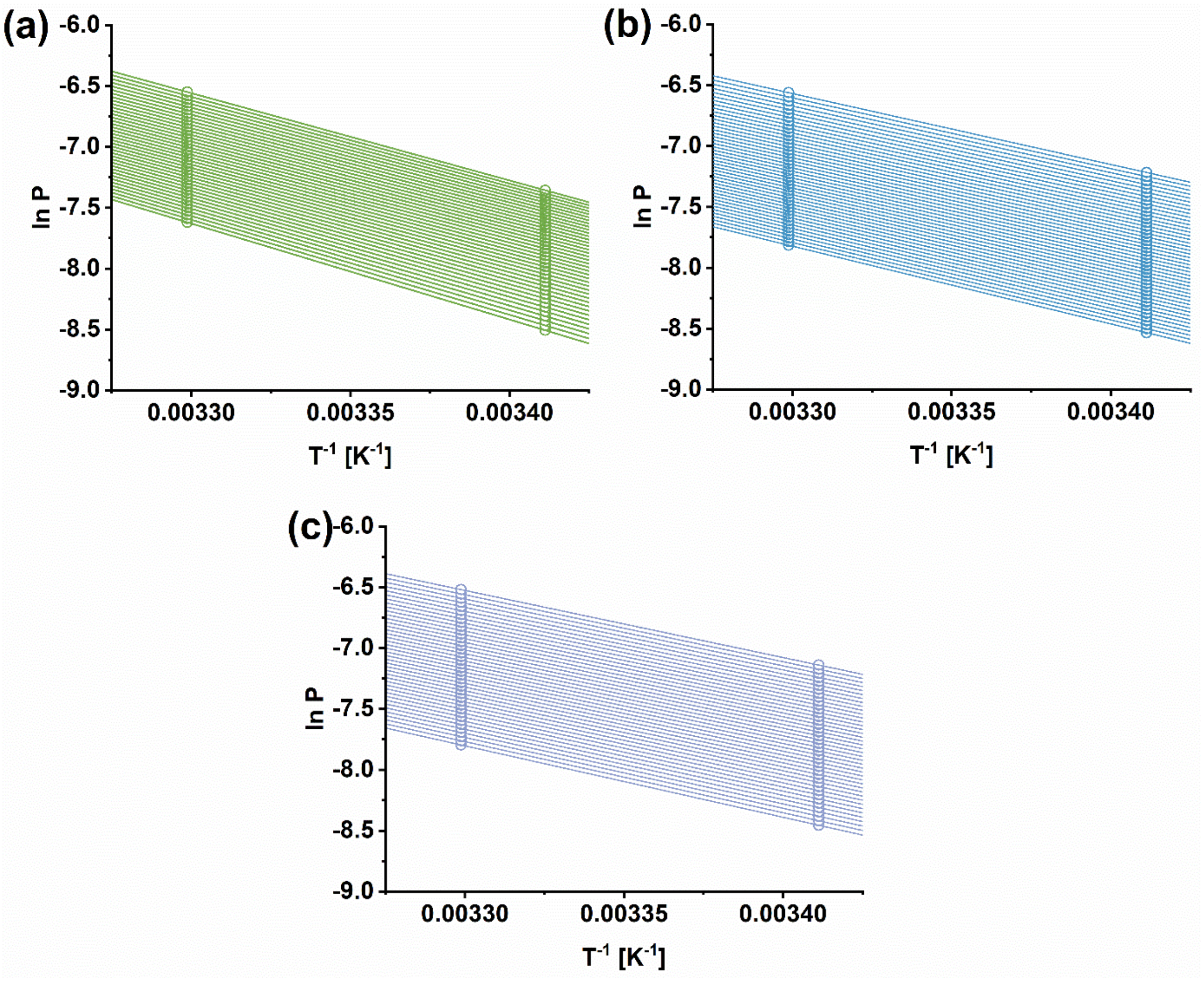

Figure S31. Isosteric plots of ln P vs T^{-1} of (a) VOFFIVE-1-Ni, (b) NbOFFIVE-1-Ni, and TaOFFIVE-1-Ni, obtained from CO_2 adsorption isotherms recorded at 293 – 303 K and 0.19 – 1.49 mbar fitted using the Langmuir-Freundlich model.

S3.5.3. H_2O sorption isotherm of NbOFFIVE-2-Ni

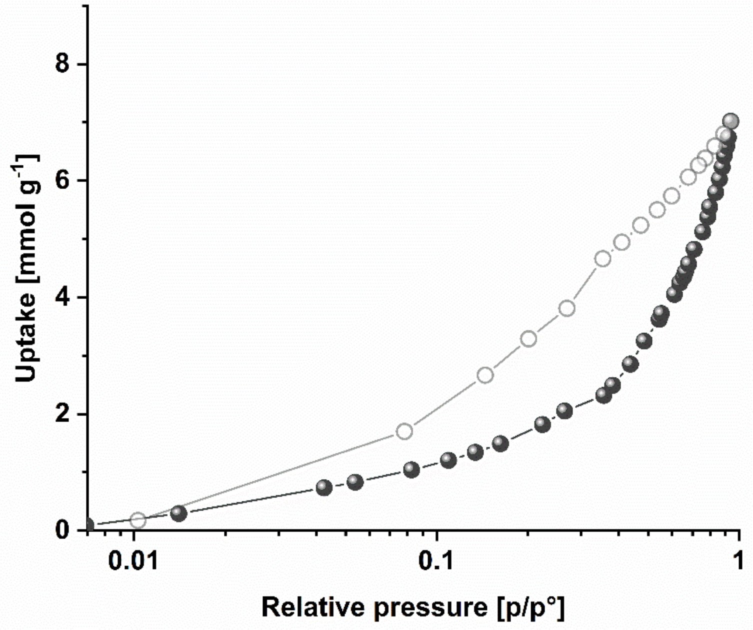

Figure S32. H_2O sorption isotherm recorded at 293 K. Filled and hollow spheres represent the adsorption and desorption branches, respectively.

S3.6. *In situ* infrared spectroscopy

Time-dependent *in situ* infrared (IR) spectra were collected on a Varian 670-IR FT-IR spectrometer (Varian Inc./Agilent Technologies, California, USA) equipped with a mercury cadmium telluride (MCT) detector. The samples were ground into fine powder and dispersed in ethanol. One drop of sample dispersion was deposited on a 16 mm circular calcium fluoride (CaF_2) pellet and allowed to dry. The CaF_2 pellet was then placed into a homemade IR transmission cell. The pellet was activated inside the IR transmission cell at a low pressure (1×10^{-4} Pa) at 383 K. A proportional-integral-derivative controller was used to maintain the temperature during the activation and CO_2 sorption experiments (303 K). Pure gas spectra were recorded separately without a sample in the IR transmission cell at the same pressure range used for the CO_2 sorption experiments (1000 ppm equivalent).

Time-resolved CO_2 uptake data were obtained from time-dependent IR spectra. The IR spectra were collected by rapidly introducing CO_2 gas (>99.999%, provided by Linde gas company), into the manifold and subsequently collecting IR spectra every 0.25 s for up to 60 min.

The single beam absorption spectra of the sorbent without CO_2 (I_0) and with CO_2 (I) were used to calculate the absorption spectra for CO_2 adsorbed on the sorbent using the relationship $A = \log_{10}(I_0/I)$. This calculation was carried out for every time point.

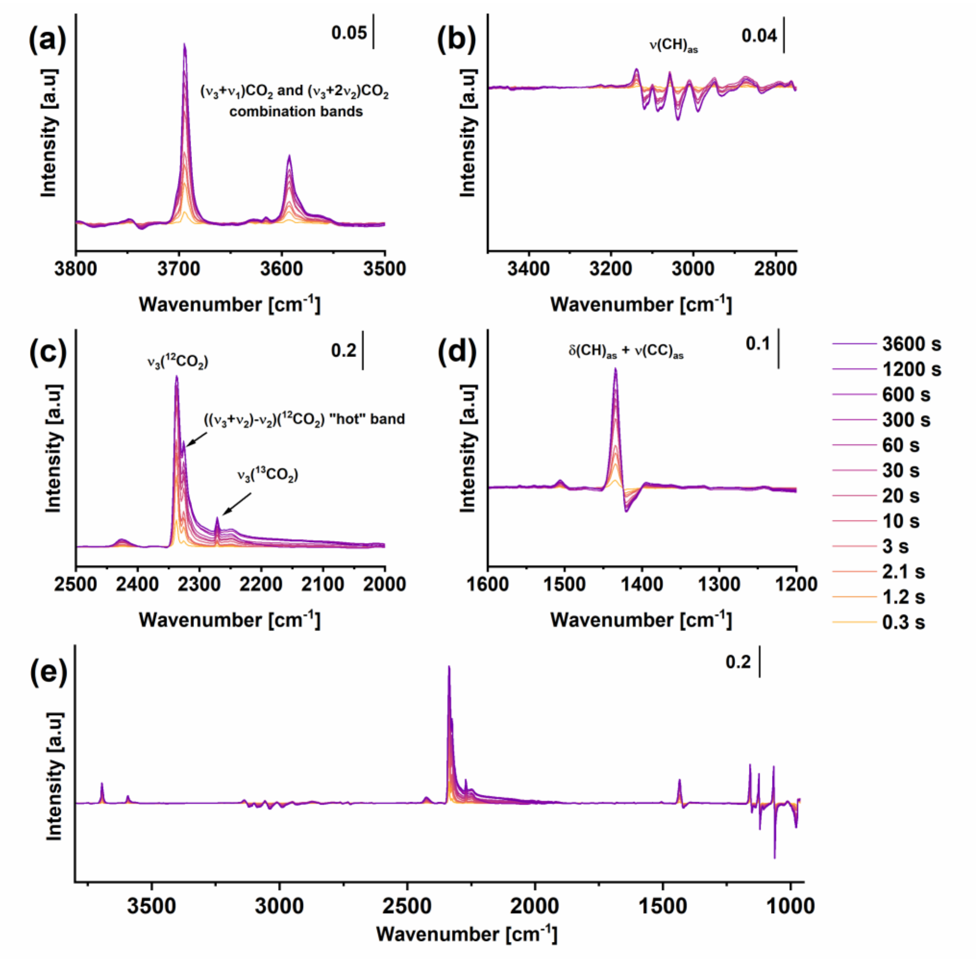

Figure S33. Time-dependent *in situ* infrared spectra showing the effects of CO_2 adsorption on VOFFIVE-1-Ni. (a – d) close-up of specific regions and (e) full spectrum. All the spectra were monitored by time progression from 0.3 to 3600 s.

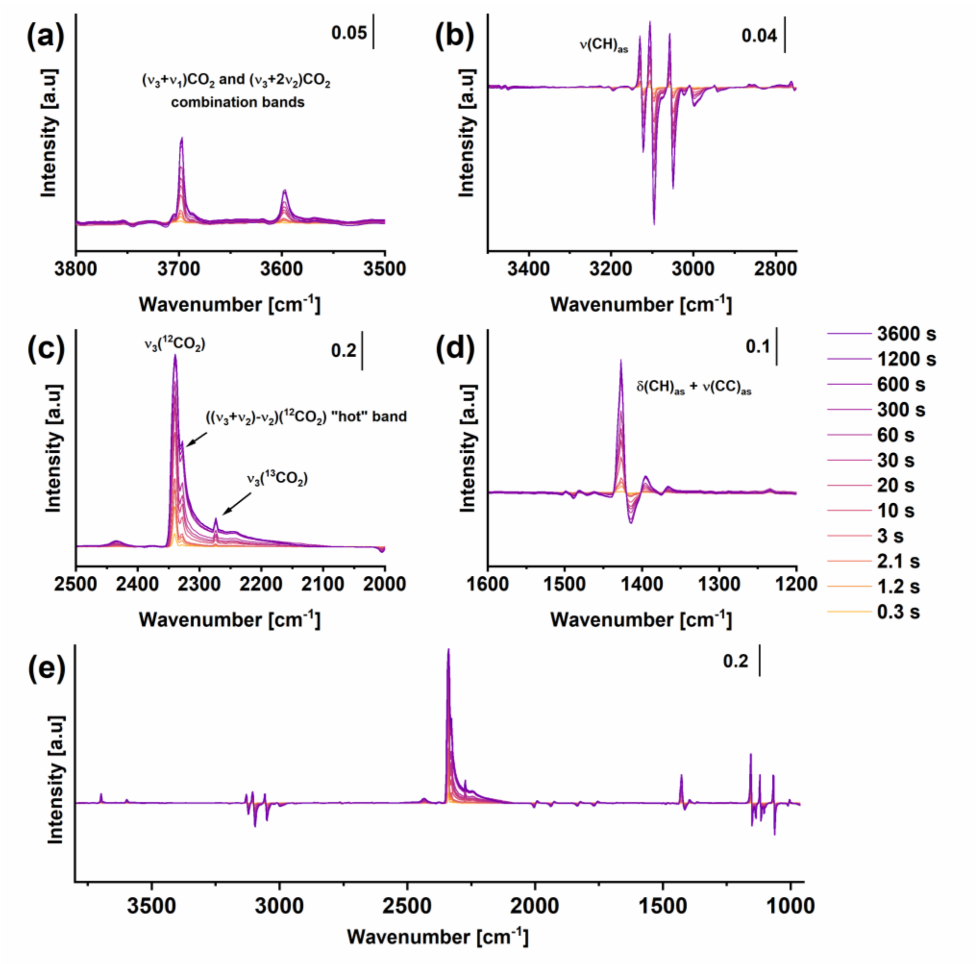

Figure S34. Time-dependent *in situ* infrared spectra showing the effects of CO_2 adsorption on NbOFFIVE-1-Ni. (a – d) close-up of specific regions and (e) full spectrum. All the spectra were monitored by time progression from 0.3 to 3600 s.

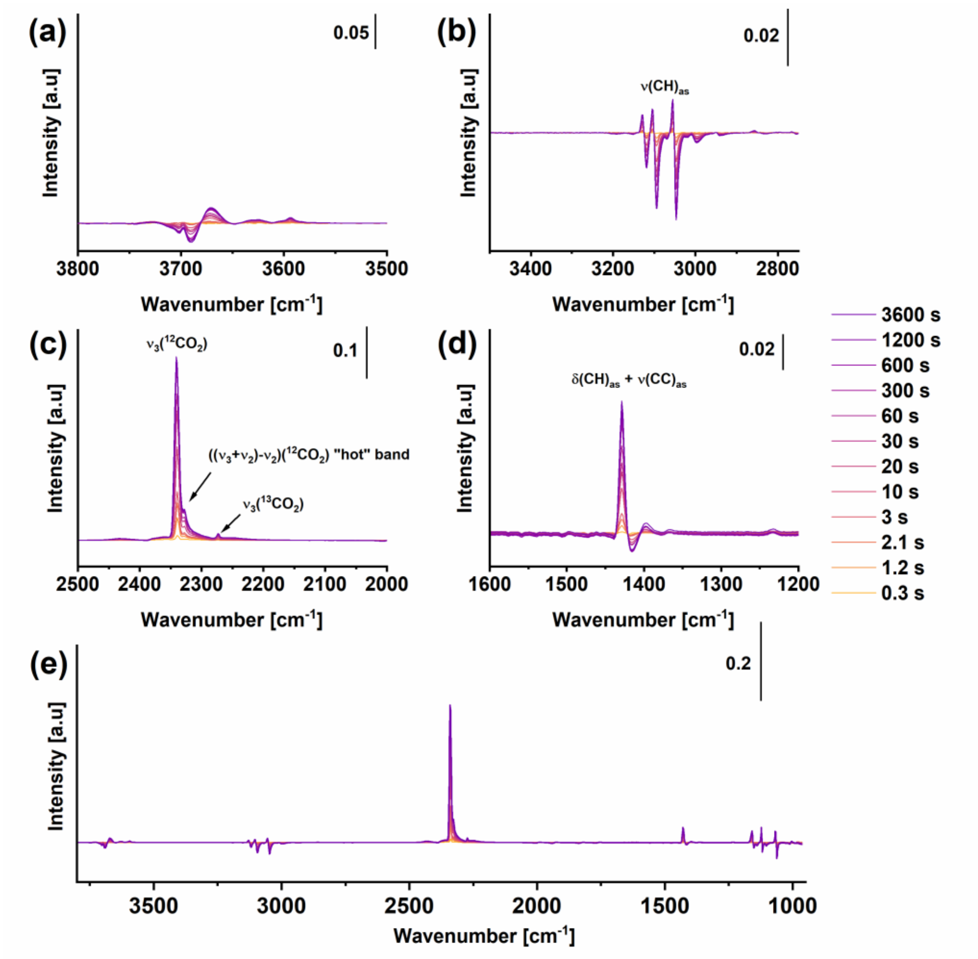

Figure S35. Time-dependent *in situ* infrared spectra showing the effects of CO_2 adsorption on TaOFFIVE-1-Ni. (a – d) close-up of specific regions and (e) full spectrum. All the spectra were monitored by time progression from 0.3 to 3600 s.

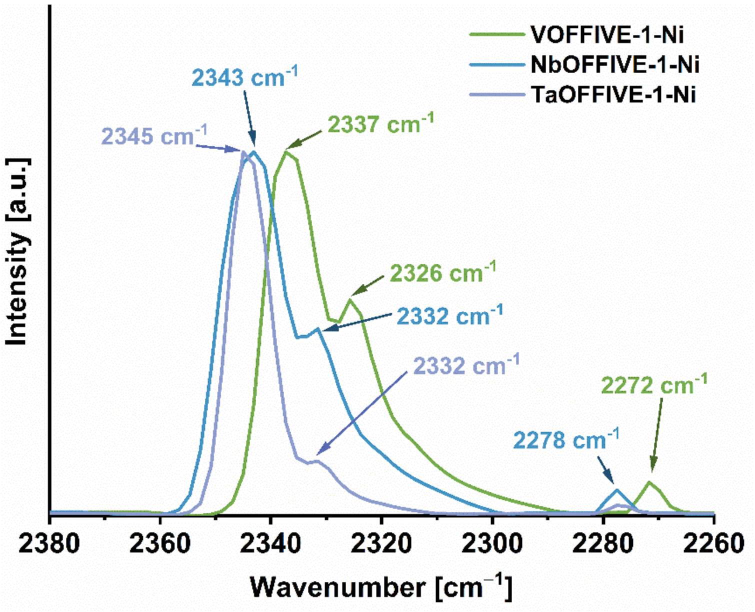

Figure S36. *In situ* infrared IR spectra showing the $\nu_3(CO_2)$-region recorded after 3600 s of CO_2 adsorption on VOFFIVE-1-Ni, NbOFFIVE-1-Ni, and TaOFFIVE-1-Ni.

S3.7. Scanning electron microscopy images

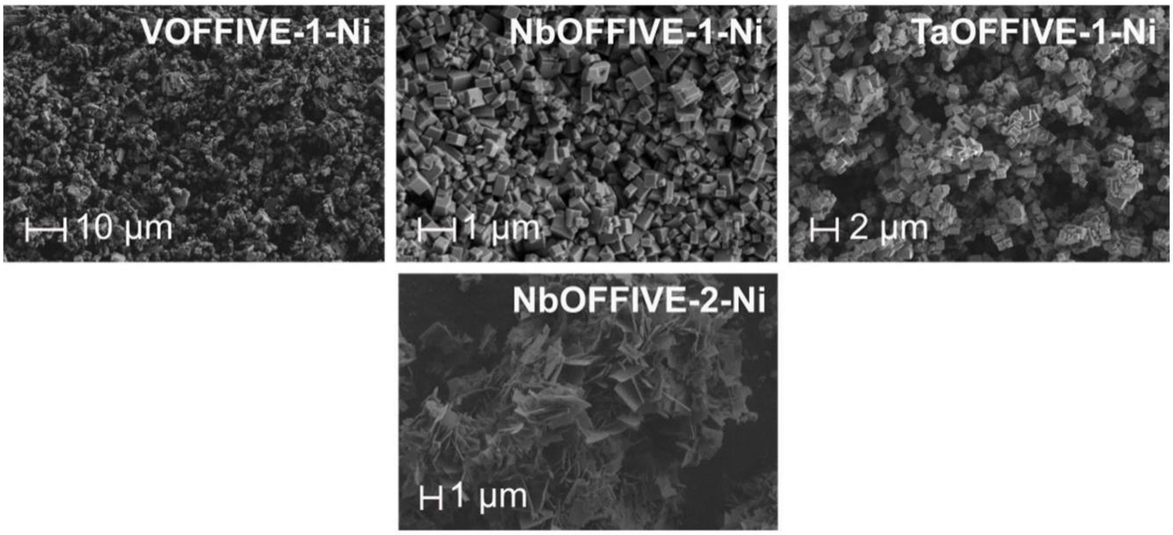

Figure S37. Scanning electron microscopy (SEM) images of isoreticular VOFFIVE-1-Ni, NbOFFIVE-1-Ni, TaOFFIVE-1-Ni, and NbOFFIVE-2-Ni.

S3.8. CO_2 adsorption kinetics

The CO_2 diffusional time constants (D/r^2) were calculated using micropore diffusional controlled transient diffusion equation for a spherical adsorbent particle[12]:

$$\frac{m_t}{m_\infty} = 1 - \frac{6}{\sqrt{\pi}} \sum_{n=1}^{\infty} \frac{1}{n^2} exp\left(-\frac{n^2\pi^2 Dt}{r^2}\right) \qquad \text{Equation 3}$$

Which in the short-time region can be reduced and expressed as:

$$\frac{m_t}{m_e} \approx \frac{6}{\sqrt{\pi}}\sqrt{\frac{Dt}{r^2}} \qquad \text{Equation 4}$$

Where m_t/m_e denotes the fractional uptake, t [s] time, and D/r^2 [s^{-1}] the diffusional time constant. D/r^2 can be calculated from a plot of m_t/m_e versus $t^{1/2}$ in which the slope in the short-time region equals:

$$Slope = \frac{6}{\sqrt{\pi}}\sqrt{\frac{D}{r^2}} \qquad \text{Equation 5}$$

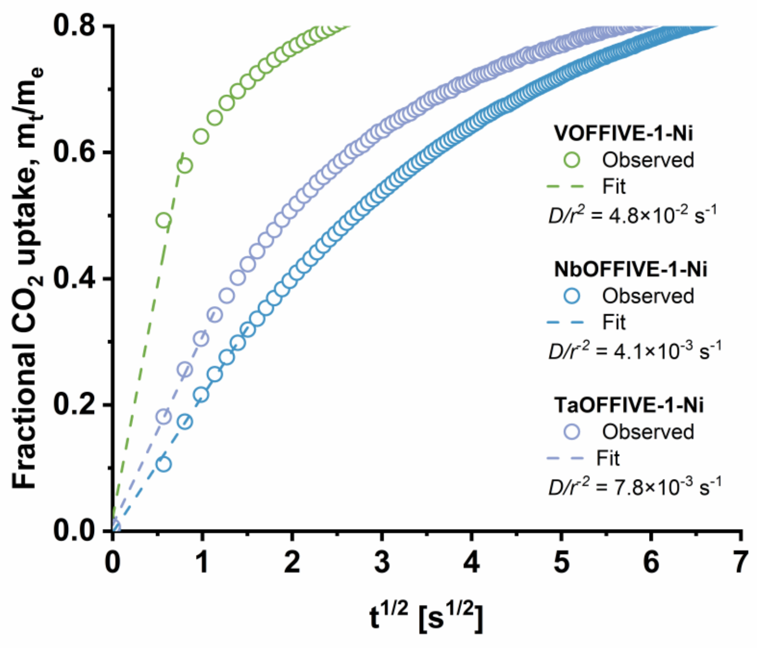

Figure S38. CO_2 profiles derived from in situ infrared data fitted using the short-time expression of the transient diffusion equation. The infrared measurements were carried out at 303 K and 1000 ppm CO_2.

Table S8. Summary of fitting parameters.

Material	Slope [$s^{-1/2}$]	Intercept	R^2
VOFFIVE-1-Ni	0.738	0.022	0.9779
NbOFFIVE-1-Ni	0.216	−0.002	0.9960
TaOFFIVE-1-Ni	0.298	0.010	0.9987

S4. References

(1) Cadiau, A.; Adil, K.; Bhatt, P. M.; Belmabkhout, Y.; Eddaoudi, M. A Metal-Organic Framework-based Splitter for Separating Propylene from Propane. *Science* **2016**, *353* (6295), 137-140.

(2) Gao, B.; Zhang, Z.; Hu, J.; Cui, J.; Chen, L.; Cui, X.; Xing, H. Efficient Separation of C4 Olefins using Tantalum Pentafluor Oxide Anion-Pillared Hybrid Microporous Material. *Chin. J. Chem. Eng.* **2022**, *42* 49-54.

(3) Cichocka, M. O.; Angstrom, J.; Wang, B.; Zou, X.; Smeets, S. High-throughput continuous rotation electron diffraction data acquisition via software automation. *Journal of Applied Crystallography* **2018**, *51* (6), 1652-1661.

(4) Kabsch, W. XDS. *Acta Crystallogr. D Biol. Crystallogr.* **2010**, *66* (Pt. 2), 125-132.

(5) Huang, Z.; Svensson Grape, E.; Li, J.; Inge, A. K.; Zou, X. 3D Electron Diffraction as an Important Technique for Structure Elucidation of Metal-Organic Frameworks and Covalent Organic Frameworks. *Coord. Chem. Rev.* **2021**, *427* 213583.

(6) Ge, M.; Wang, Y.; Carraro, F.; Liang, W.; Roostaeinia, M.; Siahrostami, S.; Proserpio, D. M.; Doonan, C.; Falcaro, P.; Zheng, H.; Zou, X.; Huang, Z. High-Throughput Electron Diffraction Reveals a Hidden Novel Metal–Organic Framework for Electrocatalysis. *Angew. Chem. Int. Ed.* **2021**, *60* (20), 11391-11397.

(7) Yang, T.; Willhammar, T.; Xu, H.; Zou, X.; Huang, Z. Single-Crystal Structure Determination of Nanosized Metal-Organic Frameworks by Three-Dimensional Electron Diffraction. *Nat. Protoc.* **2022**, *17* (10), 2389-2413.

(8) Gemmi, M.; Mugnaioli, E.; Gorelik, T. E.; Kolb, U.; Palatinus, L.; Boullay, P.; Hovmöller, S.; Abrahams, J. P. 3D Electron Diffraction: The Nanocrystallography Revolution. *ACS Cent. Sci.* **2019**, *5* (8), 1315-1329.

(9) Zhao, J. Push the Limitations of Crystal Structure Determination by 3D Electron Diffraction: From Inorganic Porous Materials to Biomolecules, Department of Materials and Environmental Chemistry, Stockholm University, Stockholm, Department of Materials and Environmental Chemistry (MMK), Stockholm University, **2021**, pp. 82

(10) Osterrieth, J. W. M.; Rampersad, J.; Madden, D.; Rampal, N.; Skoric, L.; Connolly, B.; Allendorf, M. D.; Stavila, V.; Snider, J. L.; Ameloot, R.; Marreiros, J.; Ania, C.; Azevedo, D.; Vilarrasa-Garcia, E.; Santos, B. F.; Bu, X.-H.; Chang, Z.; Bunzen, H.; Champness, N. R.; Griffin, S. L.; Chen, B.; Lin, R.-B.; Coasne, B.; Cohen, S.; Moreton, J. C.; Colón, Y. J.; Chen, L.; Clowes, R.; Coudert, F.-X.; Cui, Y.; Hou, B.; D'Alessandro, D. M.; Doheny, P. W.; Dincă, M.; Sun, C.; Doonan, C.; Huxley, M. T.; Evans, J. D.; Falcaro, P.; Ricco, R.; Farha, O.; Idrees, K. B.; Islamoglu, T.; Feng, P.; Yang, H.; Forgan, R. S.; Bara, D.; Furukawa, S.; Sanchez, E.; Gascon, J.; Telalović, S.; Ghosh, S. K.; Mukherjee, S.; Hill, M. R.; Sadiq, M. M.; Horcajada, P.; Salcedo-Abraira, P.; Kaneko, K.; Kukobat, R.; Kenvin, J.; Keskin, S.; Kitagawa, S.; Otake, K.-I.; Lively, R. P.; DeWitt, S. J. A.; Llewellyn, P.; Lotsch, B. V.; Emmerling, S. T.; Pütz, A. M.; Martí-Gastaldo, C.; Padial, N. M.; García-Martínez, J.; Linares, N.; Maspoch, D.; Suárez del Pino, J. A.; Moghadam, P.; Oktavian, R.; Morris, R. E.; Wheatley, P. S.; Navarro, J.; Petit, C.; Danaci, D.; Rosseinsky, M. J.; Katsoulidis, A. P.; Schröder, M.; Han, X.; Yang, S.; Serre, C.; Mouchaham, G.; Sholl, D. S.; Thyagarajan, R.; Siderius, D.; Q. Snurr, R.; Goncalves, R. B.; Telfer, S.; Lee, S. J.; Ting, V. P.; Rowlandson, J. L.; Uemura, T.; Iiyuka, T.; van derVeen, M. A.; Rega, D.; Van Speybroeck, V.; Rogge, S. M. J.; Lamaire, A.; Walton, K. S.; Bingel, L. W.; Wuttke, S.; Andreo, J.; Yaghi, O.; Zhang, B.; Yavuz, C. T.; Nguyen, T. S.; Zamora, F.; Montoro, C.; Zhou, H.; Kirchon, A.; Fairen-Jimenez, D. How Reproducible are Surface Areas Calculated from the BET Equation? *Adv. Mater.* **2022**, *34* (27), e2201502.

(11) Brunauer, S. The adsorption of gases and vapors, Physical Adsorption, Princeton University Press, Princeton, **1945**.

(12) Kärger, J.; Ruthven, D. M.; Theodorou, D. N. Sorption Kinetics, Diffusion in Nanoporous Materials, Wiley-VCH Verlag GmbH & Co. KGaA, Weinheim, Germany, **2012**, pp. 146-147.
